# Dietary factors and hypertension risk in West Africa: a systematic review and meta-analysis of observational studies

**DOI:** 10.1097/HJH.0000000000003499

**Published:** 2023-07-05

**Authors:** Nimisoere P. Batubo, J. Bernadette Moore, Michael A. Zulyniak

**Affiliations:** Nutritional Epidemiology Group, School of Food Science and Nutrition, University of Leeds, Leeds, UK

**Keywords:** dietary factors, hypertension, meta-analysis, systematic review, West Africa

## Abstract

**Background:**

Contrary to North America and Europe, the prevalence of hypertension is rising in West Africa. Although diet is implicated as a contributor to this trend, nutritional guidelines in West Africa are not tailored to address this concern. This study aimed to address this limitation by investigating dietary factors common to West Africa and evaluating their association with hypertension.

**Methods:**

PubMed, Scopus, Web of Science, and Medline were searched to identify studies that investigated diet and hypertension in West African adults. All meta-analyses used a generic inverse-variance random effects model, with subgroup analyses by age, BMI, and study location, and were performed in R.

**Results:**

Three thousand, two hundred ninety-eight studies were identified, of which 31 (*n* = 48 809 participants) satisfied inclusion criteria – all cross-sectional. Meta-analyses of the association between dietary factors and hypertension included dietary fat [odds ratio (OR) = 1.76; 95% confidence interval (95% CI) 1.44–2.14; *P* < 0.0001], red meat (OR = 1.51; 95% CI: 1.04–2.18; *P* = 0.03), junk-food (OR = 1.41; 95% CI: 1.19–1.67; *P* < 0.0001), dietary salt (OR = 1.25; 95% CI: 1.12–1.40; *P* < 0.0001), alcohol (OR = 1.17; 95% CI: 1.03–1.32; *P* = 0.013), and ‘fruits and vegetables’ (OR = 0.80; 95% CI: 0.24–1.17; *P* < 0.0001). Subgroup analyses suggested that ‘fruit and vegetable’ consumption is less protective in the elderly.

**Conclusion:**

High consumption of dietary salt, red meat, dietary fat, junk food, and alcohol are associated with increased odds of hypertension, whereas high fruit and vegetable appear protective. This region-specific evidence will support the development of nutritional assessment tools for clinicians, patients, and researchers aiming to reduce hypertension in West Africa.

## Graphical abstract

**Figure:**
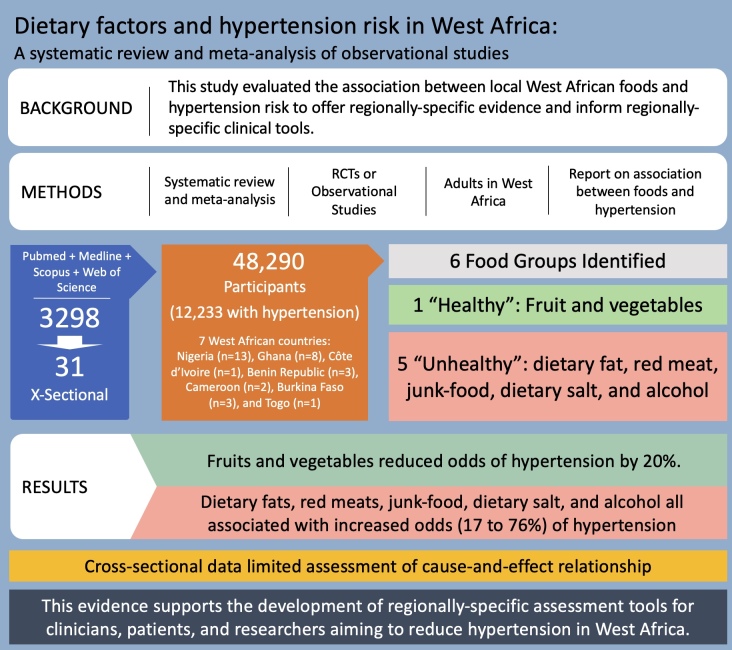

## INTRODUCTION

Hypertension, defined as a sustained elevation of blood pressure at least 140/ 90 mmHg, remains the leading preventable risk factor for cardiovascular disease and the number one cause of death globally [1]. Approximately 40% of people aged 30–79 years have hypertension, corresponding to one in three adults (∼1.3 billion in 2019), with two-thirds of cases living in low and middle-income countries, including African countries [2,3]. The most recent WHO data show that the African region has the highest prevalence of hypertension (35.5%), while the Americas have the lowest (18%) [4]. From 2010 to 2019, the UK and China experienced a decrease in hypertension rates of 5.0 and 16.3%, respectively [5,6], while in the USA, age-adjusted prevalence of hypertension among adults increased has been relatively consistent since 2009 (∼32%) [7]. Conversely, West African countries, such as Nigeria, have consistently seen an increase in hypertension rates. Recent data indicate a 15.3% increase in hypertension rates in Nigeria from 2010 to 2019 [8,9]. This rise in hypertension rates has also coincided with an increase in heart disease, stroke, and chronic kidney disease in West African countries [10,11].

This increased trend in hypertension in Nigeria and other West African countries has been partly attributed to increased unhealthy dietary practices (consumption of a diet that is high salt/sodium, low potassium consumption, unhealthy dietary fat and oils, refined sugar, alcohol, and low ‘fruit and vegetable’) and lack of physical activity [1,12–15]. Indeed, diet has been attributed to nearly one-third of all hypertension cases [16]. This positions diet as a major risk factor of interest in West Africa for reducing hypertension prevalence. In the UK, from 2003 to 2011, government and industry initiatives led to mean reductions in salt (−1.4 g/day) and increased fruit and vegetable (+0.2 portion/day) intake, which coincided with reductions in mean blood pressure (systolic = −2.7 ± 0.34 mmHg; diastolic = −1.1 ± 0.23 mmHg), stroke (−42%), and ischaemic heart disease mortality (−40%) [17]. In response, Nigeria and other West African countries outlined national nutritional guidelines to combat all noncommunicable diseases (NCDs), including hypertension [18,19]; however, the translation of these guidelines into actionable advice for clinicians for combating hypertension specifically has been a challenge, possibly because clinicians in Nigeria and other West African countries have not been provided with an effective strategy to provide regionally-specific dietary information to their patients, and the evidence used to inform West African diet recommendations is based on evidence weighted towards studies from non-African countries, which may not be translatable or applicable to manage the contribution of diet towards the rising trend of hypertension risk in Nigeria and other West African countries.

To address this, an understanding of the dietary habits and risk of hypertension in Nigeria and other West African countries is needed to confirm current recommendations and improve current dietary assessment strategies to combat hypertension. Therefore, this study aimed to undertake a systematic review and meta-analysis to provide regionally specific evidence relating to the association between dietary factors and hypertension risk in West Africa that can be used to inform the development of nutritional assessment tools for clinicians, patients, and researchers and contribute towards reducing the prevalence of hypertension in West Africa.

## MATERIALS AND METHODS

First, we performed a pilot study and evaluated the summaries of the articles we identified through our search on PubMed and which fulfilled the eligibility criteria. This step enabled us to obtain an outline of the key aspects that ought to be encompassed in the review and helped us to prepare the protocol for the systematic review and meta-analysis.

### Study design and registration

The strategy for the systematic review and meta-analysis was predefined with PROSPERO (CRD42022339736), which followed the guidelines for the Meta-analyses of observational epidemiological studies [20], and Preferred Reporting Items for Systematic review and Meta-Analysis (PRISMA) 2015 [21].

### Literature search strategy

The systematic literature search was performed using a structured PICO (population, intervention, comparison, and outcome) framework and Medical Subject Headings (MESH) indexing with key terms (and synonyms thereof). The PICO elements included the targeted West African nations (P), high dietary exposure (I), low dietary exposure (C), and hypertension/high blood pressure (O). A comprehensive search was conducted across PubMed, Ovid Medline, Scopus, and Web of Science, to identify studies published between January 2000 and February 2023. The full search strategy used for PubMed is given in Table S1 in the supplementary material. To ensure that no relevant studies were missed, the citation lists of included studies were searched to identify additional relevant studies that meet the inclusion criteria.

### Outcome measures

Four of the most prevalent adverse outcomes of blood pressure were chosen as outcomes of interest for this review and meta-analysis. These included high SBP, high DBP, high mean arterial blood pressure, and high pulse pressure.

### Study selection

Observational studies with summary data [odds ratios (ORs), relative risks (RRs), or hazard ratios with corresponding 95% confidence intervals (95% CIs), *P*-value, means with standard deviation or standard error] that addressed any dietary intervention on hypertension or high blood pressure in healthy West African populations with participants aged at least 18 years were included in the study. Studies were excluded if the data were hypertensive individuals alone, animal and cells studies, non-West African populations, noninvestigative studies (such as editorials, reviews, and conference abstracts), pregnancy studies, cardiovascular diseases (such as stroke, myocardial infarction) or studies in cancer populations, studies that are not related to diet, food or nutrition, studies involving only alcohol, smoking, or biomarkers of diet. The screening of ‘title and abstract’ and ‘full text’ was performed in duplicate in DistillerSR (v 2.35; DistillerSR Inc.) [22].

### Data collection and extraction

Using a standardized form, data from each study were independently extracted by two reviewers for all variables. Dietary factors identified from the observational studies were analysed separately. Information extracted from each study included study details (first author, year of publication, geographic location, number of participants, number of cases, study design), demographic information related to confounding (e.g. age, sex, country, health status), anthropometric data (e.g. BMI), methods of quantifying and measuring diet, types of measurements and the studies outcomes (blood pressure), univariable or multivariable effect estimate (RRs, hazard ratios, or ORs including the corresponding CIs), and adjusted covariates. Where multiple analytical models were provided, effect estimates from fully adjusted were extracted. When the risk estimates for participants were reported separately for men and women in a study or for similar food groups in this same population, the ORs were combined using a fixed-effect model [23].

### Quality assessment

To assess the quality of the studies, a modified Newcastle-Ottawa Scale (NOS) adapted for cross-sectional studies was used to evaluate the study quality of cross-sectional studies of the included studies [24]. The scale assesses the selection of participants, comparability of groups, and assessment of outcomes. A total of 0–9 was assigned to each study based on the number of stars, with a higher score indicating higher quality. The risk of bias assessment was carried out independently by two researchers. Disagreements between researchers were resolved by a third researcher.

### Data synthesis and analysis

To derive summary ORs and 95% CIs, we applied a meta-analysis to investigate the associations of the dietary factors of the highest consumers compared with the lowest consumers using the generic inverse-variance weighted random-effects model. Using an inverse variance method, we calculated the standard errors for the logarithm OR of each study. This was, in turn, considered the estimated variance of the logarithm OR. The DerSimonian-Laird estimator was used to estimate the between-study variance (τ^2^) in the random-effects model [25]. In the meta-analyses with less than five studies, the Hartung- Knapp-Sidik-Jonkman (HKSJ) random effects model was also used. In addition, where three studies or less were available for meta-analysis, a fixed effects model was also performed to retain power, and Bonferroni correction was applied where necessary [26–28].

The heterogeneity between studies was evaluated using Cochran's Q statistics [29] and the *I*^2^ statistic, with uncertainty intervals, where *I*^2^ more than 50% indicated significant statistical heterogeneity [23,30,31]. Subgroup analyses and meta-regression were conducted to explore potential sources of heterogeneity by prespecified characteristics and hypertension confounders: BMI (BMI < 25 vs. ≥ 25 kg/m^2^), the mean age of the study population (mean age: < 50 vs. ≥ 50 years), and the study location. Sensitivity analysis was performed when *I*^2^ more than 50% to estimate the influence of a single study on the overall pooled results by systematically omitting a single study and observing its influence on the overall effect size.

Publication bias was done by funnel plots inspection, where at least 10 studies were available for a single meta-analysis. Where publication bias was visibly apparent, further analyses (Egger's regression and rank correlation test) were undertaken to estimate the effect of reporting bias on study results using the standard error of the observed outcomes as predictors, which are used to check for funnel plot asymmetry [32,33]. The trim-and-fill method was also used to correct publication bias if detected. Where no significant effects were found, posthoc power analyses were undertaken using fixed-effects (τ2 = 0) or random-effects (τ2>0) methods. Power at least 80% was considered adequate [23]. All statistical analyses were performed using meta-package (version 6.0-0) [34] in R [35].

## RESULTS

### Literature search

**FIGURE 1:**
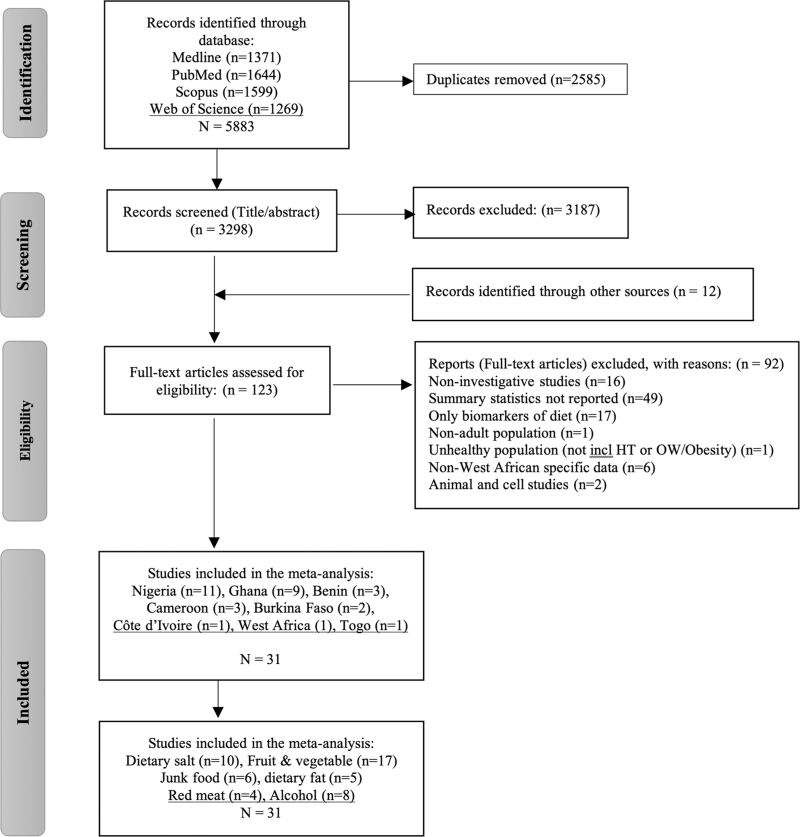
PRISMA flowchart. A total of 5883 studies were identified through database searches, and an additional 12 studies were identified through manual searches. After removing duplicates and screening titles and abstracts, 123 studies were assessed for eligibility. Of these, 31 studies met the inclusion criteria and were included in the meta-analysis. HT, hypertension; incl, including; OW, overweight.

The literature search and study selection processes are summarized in the PRISMA flow diagram in Fig. 1. A total of 5883 records were identified from the initial database search (Medline = 1371, PubMed = 1644, Scopus = 1599, Web of Science = 1269). After removing duplicates (*n* = 2585 records) and those that did not fit the inclusion criteria (*n* = 3187), 123 articles were retrieved for full-text screening, from which 31 studies were included in the meta-analysis.

### Study characteristics

In total, 31 cross-sectional studies conducted in West Africa were included in the meta-analysis—Nigeria (*n* = 13), Ghana (*n* = 8), Côte d’Ivoire (*n* = 1), Benin Republic (*n* = 3), Cameroon (*n* = 2), Burkina Faso (*n* = 3), and Togo (*n* = 1)—with an age range of 20–55 years (mean age = 41 years) and an average BMI of 25.9 kg/m^2^ (Table 1). A total of 48 290 participants were included in the studies (of which 12 223 were diagnosed with hypertension), with sample sizes ranging from 134 to 9367. The studies covered a range of dietary factors, with 17 reporting on fruit and vegetable intake (i.e. ‘healthy foods’) and numerous ‘unhealthy foods’, including six on junk food (fried and fast food), four on red meat, five on dietary fats (fatty food), 10 on dietary salt, and eight on alcohol consumption.

**TABLE 1:** Characteristics of the included studies investigating the association between dietary factors and hypertension risk in West Africa

									Effect sizes
S/N	Ref.	Study location	Dietary assessment instrument used (specific items queried)	Mean age (years)	BMI (kg/m^2^)	Sample size	Cases	Dietary factor	OR (95% CI)
1	Anto *et al.*[53]	Ghana	Not published or validated structured questionnaire (Fast food, alcohol^a^)	44	29	527	204	Fast food, alcohol^a^	FF = 2.43 (1.18–5.02)
2	Sackou *et al.*[41]	Côte d’Ivoire	WHO STEP survey (FV, and alcohol only)	34	30	360	67	Fruit & vegetables, alcohol	FV = 0.87 (0.22–3.45) Alcohol = 0.82 (0.47–1.41)
3	Ugwuja *et al.*[54]	Nigeria	Not published or validated structured questionnaire (Red meat, fish, ^a^alcohol)	42	24	271	62	Red meat, fish, ^a^alcohol	Meat = 2.55 (1.11–5.83)
4	Oguoma *et al.*[42]	Nigeria	WHO STEP survey (FV, alcohol, oil^a^)	40		417	34	Fruit & vegetables, alcohol, oil^a^	FV = 0.94 (0.90–0.98) Alcohol = 1.85 (1.18–2.88)
5	Olawuyi [43]	Nigeria	WHO STEP survey (FV, and Binge alcohol only)	43	27	606	201	Fruit & Vegetables, Binge drinking	FV = 0.91 (0.48–1.72), FV = 1.10 (0.58–2.08) Binge = 2.98 (1.00–8.91)
6	Acheampong *et al.*[55]	Ghana	Not published or validated Structured questionnaire (salt, alcohol, fruit, and fast food)	50	28	216	73	Salt, Fruit, Fast food, alcohol	FV = 0.32 (0.11-- 0.93) Salt = 1.34 (0.64–2.81) FF = 1.45 (0.69–3.05) Alcohol = 1.55 (0.77–3.12)
7	Obasohan *et al.*[36]	Nigeria	Food and Agricultural organisation FFQ (FAO-FFQ; validated) (fast food only)	41	26	476	208	Fast Food	FF = 1.28 (0.80–2.03)
8	Menyanu *et al.*[44]	Ghana	WHO STEP survey (salt only)	58	26	4675	1444	salt	Salt = 1.11 (0.95–1.30)
9	Desormais *et al.*[45]	Benin	WHO STEP survey (FV, salt, and alcohol)	43	24	1777	584	Fruit & vegetable Salt, Alcohol	FV = 0.97 (0.69–1.36) Salt = 1.23 (1.00–1.50) Alcohol = 1.19–0.94–1.53)
10	Ayogu and Ezeh [56]	Nigeria	Not published or validated Structured questionnaire (fruit, vegetable, nut, legume, and alcohol^b^)	42	27	517	195	Fruit, vegetable, nut, legume, alcohol^b^	FV = 0.77 (0.54–1.09) F = 0.47 (0.19–1.15) V = 0.63 (0.34–1.18) Nut = 0.61 (0.32–1.16) Legume = 0.69 (0.35–1.36)
11	Ofili *et al.*[57]	Nigeria	Not published or validated structured questionnaire (salt, fat, and alcohol)	53	25	134	59	Salt, fat, alcohol	Salt = 1.59 (0.79–3.20) Fat = 1.60 (0.54–4.75) Alcohol = 1.10 (0.55–2.20)
12	Nkondjock [37]	Cameroon	Validated FFQ based on International Network of Food Data System and U.S. Department of Agriculture National Nutrition Database (fruit and vegetable, meat, ^a^[tubers, doughnuts, fish & seafood, legumes, spices and herbs, white bread, whole grains, rice, pasta, soft drinks, coffee, tea, sugar, honey and jam, cakes and cookies, sweets, ships and popcom, poultry, Milk, alcohol oils and fats, supplements]	37	27	571	223	Fruit & vegetable, red meat	FV = 0.82 (0.20–2.19) Meat = 1.35 (0.74–2.47)
13	Pancha Mbouemboue [46]	Cameroon	WHO STEPS survey (salt and alcohol^b^).	36	30	700	143	Salt, alcohol^b^	Salt = 1.05 (0.52–2.07)
14	Chukwu *et al.*[47]	Nigeria	WHO STEP survey (FV, salt and only)	39	28	410	205	Fruit, vegetable, salt intake	FV = 0.15 (0.09–0.27) Salt = 2.50 (1.23–5.05)
15	Oladoyinbo *et al.*[48]	Nigeria	WHO STEP (adapted) survey (snack, sweets, fried food, and alcohol)	35	23	300	144	Snack, puff puff, sweet, fried foods	FF = 1.35 (0.29–1.65)
16	Ayogu and Ezeh [56]	Nigeria	Not published or validated Structured questionnaire (fruit, vegetable, nut, legume, and alcohol^b^)	42	27	517	195	Fruit, vegetable, nut, legume, alcohol^b^	FV = 0.77 (0.54–1.09) F = 0.47 (0.19–1.15) V = 0.63 (0.34–1.18) Nut = 0.61 (0.32–1.16) Legume = 0.69(0.35–1.36) ^b^Alcohol = 2.04 (0.80–5.20)
17	Ayogu and Nwodo [58]	Nigeria	Not published or validated structured questionnaire (Snacks, fried/baked food, fruits, vegetables, and alcohol^b^)	20	23	401	76	Snacks, baked food products, fruits, vegetables, alcohol^b^	FF = 3.15 (0.61–16.26) SD = 1.57 (0.62–3.93) Fruit = 0.53 (0.24–1.17)
18	Akoklannou *et al.*[49]	Benin	WHO STEP survey (FV, salt, fatty food, and alcohol)	35	24	717	238	Salt, Fatty food, Fruits and vegetables, alcohol	Salt = 1.54 (1.07–2.21) Fruit = 0.75 (0.37–1.52) Fat = 2.07 (0.94–4.54) Alcohol = 1.04 (0.83–1.30)
19	Soubeiga *et al.*[50]	Burkina Faso (Rural)	WHO STEP survey (Butter/lard/margarine/oil)	44	32	3600	553	Butter, lard, margarine, vegetable oil	Fat = 1.98 (1.22–3.22)
20	Yayehd *et al.*[59]	Togo	Not published or validated structured questionnaire (salt and alcohol only)	49	30	2002	734	Salt, alcohol	Salt = 1.40 (1.13–1.72) Alcohol = 1.21 (0.97–1.51)
21	Colette *et al.*[60]	Benin	Not published or validated structured questionnaire (salt, fruit and vegetable, and alcohol)	40	24	540	154	Salt, fat, fruit & vegetable, alcohol	Fruit = 0.83 (0.56–1.23] Salt = 3.45 (2.22–5.06) Fat = 1.81 (1.17–2.81) Alcohol = 2.00 (0.97–4.12)
22	Boakye *et al.*[61]	Ghana	Not published or validated structured questionnaire (salt, fruit, vegetables, oil, animal meat, alcohol^a^)	44	25	242	90	Salt, fruit & vegetable, oil, meat, alcohol^a^	Salt = 1.53 (1.05–2.22) Fruit = 0.66 (0.52–0.85), Veg = 0.83 (0.64–1.08), Meat = 1.40 (0.69–2.83) oil = 1.65 (1.25–2.19)
23	Owiredu [120]	Ghana	Not published or validated structured questionnaire (fruit, vegetable)	46	27	204	100	FV	FV = 0.66 (0.35–1.25) Low FV = 1.51 (0.80–2.84)
24	Dorgbetor *et al.*[63]	Ghana	Not published or validated structured questionnaire (salt)	36	24	5662	918	Salted fish	Salt = 1.06 (0.90–1.24)
25	Diendere *et al.*, 2022 [52]	Burkina Faso	WHO STEP survey (FV, and alcohol^a^)	44	24	4187	774	Fruit, vegetable	FV = 0.36 (0.15–0.83) Low FV = 2.8 1.2–6.7
26	Akpa *et al.*[13]	Nigeria	SIREN/ AWFGEN study FFQ (vegetable)	55.4	24	3215	1727	Vegetables	V = 0.96 (0.59–1.57)
27	Akpa *et al.*[13]	Ghana	SIREN/ AWFGEN study FFQ (vegetable)	53.7	26	3214	1240	Vegetables	V = 0.97 (0.77–1.23)
28	Akpa *et al.*[13]	Burkina Faso	SIREN/ AWFGEN study FFQ (vegetable)	49.8	21	2097	345	Vegetables	V = 1.09 (0.13–9.19)
29	Shokunbi *et al.*[38]	Nigeria	WHO standard 24-h dietary recall questionnaire (carbonated drink, Puff-puff, Eggs, fruits, vegetables, Beef)	20		488	145	Puff-puff, eggs, fruits, vegetables, beef	Meat = 2.87 (1.92– 4.28) FF = 1.08 (0.68; 1.71) FV = 0.71 (0.38–1.32)
30	Makinde [39]	Nigeria	Validated FFQ (meat, fruit, vegetable, processed can meat)	34	24	397	39	Meat	Meat = 0.87 (0.37–1.96)
31	Oyekale [40]	Ghana	Validated FFQ (meat)	26	24	9367	1244	Salted meat	β= 0.06, *z* = 2.03, Meat = 1.06 (1.00–1.12)
	Total			41	25.9	48 290	12 223		

β, Beta-coefficient; AWI-Gen, Africa Wits-INDEPTH partnership for Genomic; CS, cross-sectional study; FF, Fried/fast food; FFQ, Food frequency questionnaire; FV, fruit and vegetable; NA, not available; SD, Sugary drink; SIREN, Stroke Investigative Research and Educational Network; V, vegetable; *z*, *Z*-score.

a Studies did not provide data on all items within the FFQ.

b Studies did not compare low vs. high consumption.

The studies employed various dietary assessment instruments to assess dietary intake (Table 1 and S2). Eight studies used food frequency questionnaires (FFQs) to evaluate dietary intake. These FFQs included the validated Food and Agricultural Organization FFQ (FAO-FFQ), validated FFQ based on reference values from the International Network of Food Data System and the U.S. Department of Agriculture National Nutrition Database, the SIREN/AWFGEN study FFQ, and the WHO standard 24-h dietary recall questionnaire [13,36–40]. Of the 10 studies that employed the WHO Stepwise instrument, different combinations of questions to assess the intake of salt, fruits and vegetables, snacks, sweets, fried foods, fats and oils, or alcohol were used (detailed in Table 1) [41–52]. Furthermore, 12 studies used either nonpublished or validated structured questionnaires to assess dietary intake [53–63].

### Healthy foods

#### Fruit and vegetable consumption

**FIGURE 2:**
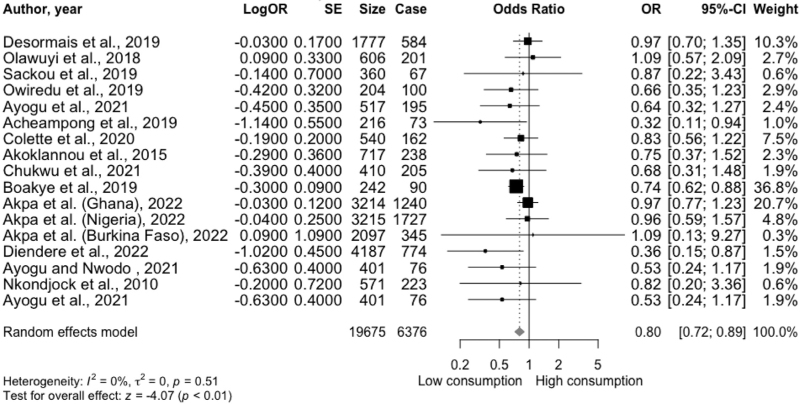
Forest plot of 17 cross-sectional studies that reported on the associaiton between fruit and vegetable consumption and hypertension in West Africa. Case, cases of hypertension; CI, confidence interval; logOR, log odds ratio; OR, odds ratio; SE, standard error; Size, sample size (number of participants).

Seventeen cross-sectional studies with 19 675 participants reported on the association between fruit and vegetable consumption with the risk of hypertension; 6376 (32.4%) adults had hypertension. The types of fruit and vegetable investigated were not specified. Mean effect sizes ranged from 0.32 to 1.09. The overall meta-analysis suggested that consuming high amounts of fruit and vegetable was associated with 20% reduced odds of hypertension (OR = 0.80; 95% CI: 0.24–1.17; *P* < 0.0001, *I*^2^ = 0%) compared with low consumers (Fig. 2). No heterogeneity or potential publication bias was evident in the funnel plot (Figure S1), rank correlation test (*P* = 0.17), or Egger's regression test (*P* = 0.18). The subgroup analysis and meta-regression indicated that while a protective effect of fruit and vegetable consumption against hypertension was observed in all West African nations, age significantly moderated the association between fruit and vegetable consumption and hypertension. It may be less pronounced in individuals aged at least 50 years. However, this may be due to fewer studies in the subgroup of individuals aged at least 50 years. Conversely, BMI and study location did not appear to modify the association (Table S4 and Figures S2 and S3).

### Unhealthy foods

#### Junk food consumption

**FIGURE 3:**
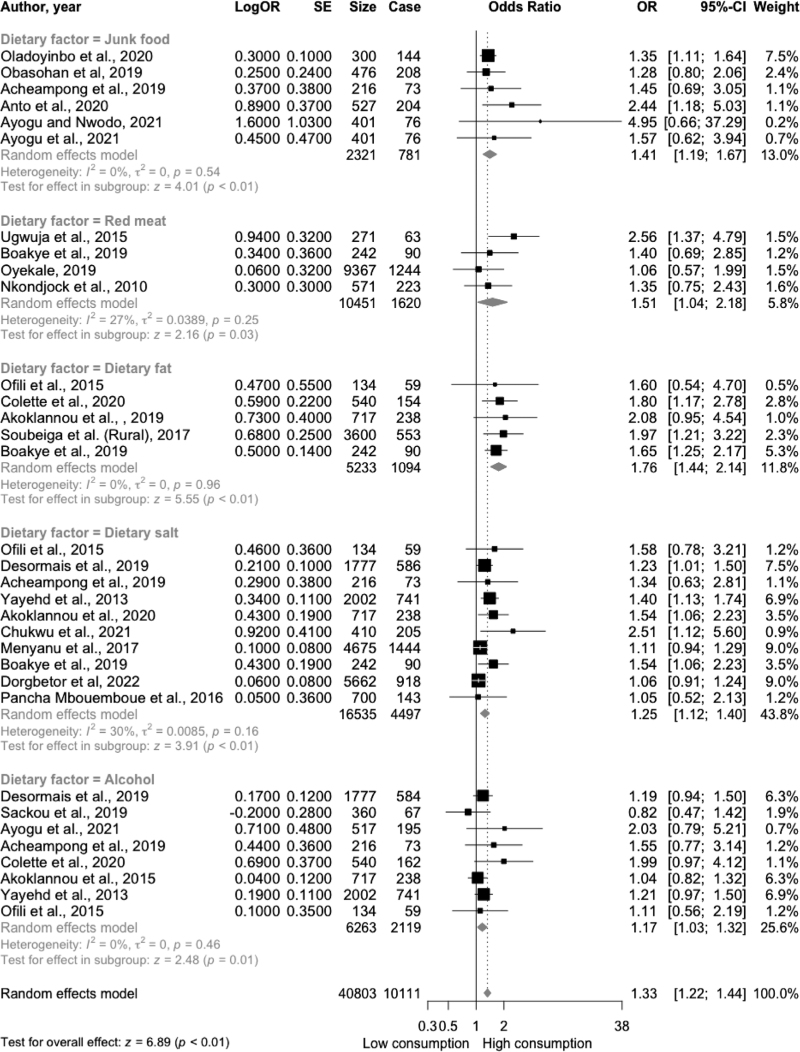
Forest plot of association between unhealthy foods (junk food, red meat, dietary fat, dietary salt, and alcohol) consumption and hypertension risk in West Africa. Case, cases of hypertension; CI, confidence interval; logOR, log odds ratio; OR, odds ratio; SE, standard error; Size, sample size (number of participants).

In the meta-analysis of the association between junk food and the risk of hypertension in West Africa, six cross-sectional studies were analysed with 2321 participants, of which 781 (33.6%) had hypertension. Junk food was defined as fried food (fried rice, fried chicken, beef, fried yam, and potatoes), fast food, fried snacks (puff-puff, fries, chips), cakes, and burgers. The mean effect sizes across individual studies ranged from 1.28 to 4.95. The meta-analysis demonstrated that consumption of high amounts of junk food was associated with increased odds of hypertension by 41% (OR = 1.41; 95% CI: 1.19–1.67; *P* < 0.0001, *I*^2^ = 0%) compared with low consumers in West Africa (Fig. 3). No heterogeneity or publication bias was observed (Figure S4), rank correlation test (*P* = 0.06), or Egger's regression test (*P* = 0.26). The subgroup analysis and meta-regression did not suggest a moderating effect by mean population age, BMI, or study location (Table S5 and Figures S5 and S6).

#### Red meat consumption

The meta-analysis of four cross-sectional studies investigating the relationship between red meat consumption and hypertension in West Africa included 10 451 participants, with 1620 (15.5%) cases of hypertension. Red meat was defined as unprocessed meats from cows, goats, pork, and sheep. The mean effect sizes from the individual studies ranged from 1.06 to 2.56. The meta-analysis suggested that high red meat consumption was associated with a 51% increase in the odds of hypertension (OR = 1.51; 95% CI: 1.04–2.18; *P* = 0.03, *I*^2^ = 27%) compared with low consumers with a moderate amount of heterogeneity (Fig. 3). No heterogeneity or publication bias was observed (Figure S7), rank correlation test (*P* = 0.72), or Egger's regression test (*P* = 1.00). Subgroup analysis and meta-regression did not suggest moderating effect of mean age, BMI, and study location on the association between the consumption of red meat and hypertension (Table S6 and Figures S8 and S9).

#### Dietary fat consumption

The meta-analysis of five cross-sectional studies examining the association between dietary fat and hypertension in West Africa included 5233 participants, including 1094 (20.9%) cases of hypertension. Dietary fat was defined as saturated oil, coconut oil, palm and palm oil, butter, lard, margarine, and groundnut oil. Effect sizes from the individual studies ranged from 1.60 to 2.08. The meta-analysis demonstrated that high dietary fat consumption was associated with 76% higher odds of hypertension (OR = 1.76; 95% CI: 1.44–2.14; *P* < 0.0001, *I*^2^ = 0%) compared with low consumers (Fig. 3). No heterogeneity or publication bias was demonstrated by the funnel plot (Figure S10), Egger's regression test (*P* = 0.89), and Begg's test (*P* = 0.48) in the meta-analysis. Subgroup analysis and meta-regression did not suggest a moderating effect on the mean population age, BMI, or study location (Table S7 and Figures S11 and S12).

#### Dietary salt consumption

A total of 10 cross-sectional studies (*n* = 16 535 participants) reported on the association between dietary salt and hypertension risk; 4497 (27.2%) adults had hypertension. Mean effect sizes ranged from 1.05 to 2.51. The analysis revealed that high consumption of dietary salt increased the odds of hypertension by 25% in West Africa (OR = 1.25; 95% CI: 1.12–1.40; *P* < 0.0001, *I*^2^ = 30%) compared with low consumers with a moderate amount of heterogeneity (Fig. 3). No potential publication bias was evident in the funnel plot (Figure S13), rank correlation test (*P* = 0.05), or Egger's regression test (*P* = 0.31). Subgroup analysis and meta-regression suggested that mean age, BMI, and study location did not seem to modify the association, as shown in Table S8 and Figures S14 and S15.

#### Alcohol consumption

A total of eight cross-sectional studies [*n* = 6263 participants, including 2119 (33.8%) cases of hypertension] reported on the association between alcohol and odds of hypertension were included in the meta-analysis, with effect sizes ranging from 0.82 to 2.03. Compared with low consumers, the meta-analysis demonstrated that high consumption of alcohol increased the odds of hypertension by 17% (OR = 1.17, 95% CI: 1.03–1.32; *P* = 0.013, *I*^2^ = 0%) (Fig. 3). No heterogeneity or publication bias was demonstrated by the funnel plot (Figure S16), Egger's regression test (*P* = 0.46), and rank correlation test (*P* = 0.17) in the meta-analysis. Subgroup analysis and meta-regression did not suggest a moderating effect on the mean population age, BMI, or study location (Table S9 and Figures S17 and S18).

### Data quality

The quality was assessed using a modified NOS for cross-sectional studies [24]. The criteria for allocating stars (out of a total of nine stars) awarded to each study according to this NOS criteria can be found in Table S3. The 30 studies included in the meta-analysis were rated with high quality (score 7–9) and a low bias risk.

## DISCUSSION

This systematic review and meta-analysis provide regional-specific evidence to support nutritional guidelines and the development of prevention strategies and tools to reduce the prevalence of hypertension in West Africa. Thirty-one cross-sectional studies with 48 290 adults in West Africa (12 223 cases of hypertension) were meta-analysed to investigate the association between common dietary factors (such as fruit and vegetables, dietary salt, junk food, red meat, dietary fat, and alcohol) and hypertension. Remarkably, this is the first meta-analysis to synthesize and analyse the impact of dietary factors on hypertension in adults living in West Africa. The results suggest that high consumption of dietary fat, red meat, junk food, dietary salt, and alcohol is associated with increased odds of hypertension, while high fruit and vegetable consumption is protective. Sensitivity analyses within all dietary factors indicate robust and consistent associations.

### Healthy foods

#### Fruits and vegetables

For single ‘healthy’ food available for analysis, fruit and vegetables, we report that high fruit and vegetable intake is associated with decreased odds of hypertension. This is consistent with previous meta-analyses conducted in other regions of the world, such as those conducted by Wu *et al.*[64] and Schwingshackl *et al.*[65]. Schwingshackl *et al.* [65] reported a decrease in hypertension risk for high vegetable (RR = 0.96; 95% CI: 0.91–1.01) and fruit intake (RR: 0.93; 95% CI: 0.87–1.00) based on even cohort studies with 94 772 incident cases of hypertension [65], while Wu *et al.* [64] found a decreased hypertension risk based on six cohort studies (RR = 0.87; 95% CI: 0.79–0.95) [64]. Although both studies reported some heterogeneity, likely due to differences in study locations and cultures, the absence of major heterogeneity in our analysis suggests that the associations are relatively consistent across West African nations. However, we did observe that the protective effects of fruits and vegetables may be less pronounced in elderly individuals, which is interesting and warrants further investigation. The results of our meta-analysis agree with biochemical evidence that reports the beneficial effects of phytochemicals, vitamins, minerals, and fibres (which are rich in fruit and vegetables) on vascular function and reduced risk of hypertension [66–76].

### Unhealthy foods

Numerous food groups were identified that have previously been negatively associated with hypertension (i.e. unhealthy foods): junk food, red meat, dietary fats, dietary salt, and alcohol.

#### Junk food

On the basis of six cross-sectional studies with 2321 participants, we report that high consumption of junk food is associated with increased odds of hypertension in West African adults. This finding agrees with biochemical evidence that has linked junk foods, which often contain high amounts of saturated and trans fats, sodium, and by-products of oxidation, which can induce increased blood pressure in different mechanisms [77–83]. In addition, our work agrees with previous work from outside West Africa. A cross-sectional study in Korea reported that incremental increases in fried food consumption associated with elevated blood pressure in men (OR = 1.62; 95% CI: 1.11–2.37; *P*_trend_ = 0.045) and (OR = 2.20; 95% CI: 1.21–4.00; *P*_trend_ = 0.040) women [84], while a meta-analysis of 11 studies (eight cohort and three cross-sectional studies) involving 222 544 participants reported that fried food increased risk of hypertension compared with low consumers [85]. The absence of heterogeneity in our meta-analysis of the association between junk food and hypertension in West Africa suggests that the findings are robust and not influenced by factors such as study design, population characteristics, or other potential sources of variation. However, it is important to note that the number of studies included in the meta-analysis is relatively small.

#### Red meat

Our meta-analysis contributes to the growing body of evidence on the association between red meat consumption and hypertension. We found that high consumption of red meat was associated with increased odds of hypertension in West African adults. This is consistent with previous meta-analyses involving seven cohort studies with 97 745 hypertension cases, albeit with higher heterogeneity (RR = 1.15; 95% CI: 1.02–1.28; *I*^2^ = 84%) [37]. Similarly, Zhang *et al.*[86] conducted a meta-analysis of nine cohort studies and reported that high consumption of red meat was associated with an increased risk of hypertension (RR = 1.22, 95% CI: 1.11–1.35) and substantial heterogeneity (*I*^2^ = 75%). Our meta-analysis observed less heterogeneity with the confounders examined not significantly modifying the observed association. The biological effect of high meat consumption on hypertension is supported by the components found in meat, such as saturated fat, cholesterol, high sodium levels in processed meat, haem iron, and substances formed during cooking or processing of meat, such as heterocyclic amines, advanced glycosylation end-products, acrylamides, and trimethylamine-N-oxide [87–95]. These components can affect blood lipids and lead to insulin resistance and inflammation, ultimately increasing the risk of hypertension.

#### Dietary fats

Our meta-analysis of five cross-sectional studies supports this evidence and indicates that in West Africa, high consumption of dietary fat is associated with increased odds of hypertension. This agrees with prospective cohort studies that have reported increased odds of hypertension (up to 40%) in population subgroups that consume high quantities of dietary fat [81,96]. Although our analysis was unable to differentiate between saturated and unsaturated fats, traditional cooking methods in West Africa typically involve the use of palm and palm kernel oils, groundnut oil, coconut oil, butter, and animal fat, which are all high in saturated and trans fats, which are most strongly associated with hypertension risk [49,50,57,81,97]. This aligns with biochemical and pathophysiological evidence that suggests that diets high in saturated and trans-fats can lead to elevated levels of blood LDL-cholesterol and triglycerides, the development of atheroma in blood vessel walls, oxidative stress, inflammation of the vessel walls, reduced vascular elasticity, and increased vascular resistance, ultimately leading to hypertension [89,98–100].

#### Dietary salt

Our meta-analysis suggests that higher consumers of dietary salt in West Africa are more likely to be hypertensive compared with limited salt consumers. Our findings are consistent with previous meta-analyses that report an increased risk of hypertension among individuals with a high dietary salt intake that live in rural and urban populations of low to middle-income countries [101], and an almost four-fold increase in odds of hypertension among 23 studies in China [102]. Despite some heterogeneity between the studies we analysed, this was not driven by study differences in age, BMI, and study location. Overall, our results align with the posited pathophysiology effects of salt/sodium on hypertension through its effect on renal salt and water retention, elevated plasma sodium levels, and increased salt sensitivity, which leads to the expansion of extracellular fluid volume, microvascular endothelial inflammation, and structural changes in autonomic and small resistant arteries, and increased systemic peripheral resistance and functional abnormalities of the cardiovascular system and hypertension [82,103–108].

#### Alcohol

Finally, our meta-analysis found that high alcohol consumption, exceeding two drinks per day for men and one drink per day for women, is associated with an increased odds of hypertension in West Africa. This finding is consistent with previous studies conducted outside West Africa, which reported an unfavourable association between alcohol consumption and hypertension. A meta-analysis of 20 cohort studies (*n* = 361 254 participants and 90 160 incident cases of hypertension) reported that alcohol consumption increases the risk of hypertension between men (RR = 1.19; 95% CI: 1.07–1.31) and women (RR = 1.42; 95% CI: 1.22–1.66) who drank more than two drinks per day in comparison with abstainers with a significant amount of heterogeneity [109]. The absence of heterogeneity or publication bias and a lack of moderating effects of mean population age, BMI, or study location on the association suggests the robustness of the association across West African nations. Pathophysiological evidence supports our findings, as alcohol stimulates renin release, which produces angiotensin II, a potent vasoconstrictor, and aldosterone increases sodium retention and water reabsorption [110,111]. Furthermore, alcohol metabolism produces reactive oxygen species (ROS) and reactive nitrogen species (RNS), which can lead to oxidative damage to the endothelium and vascular smooth muscle, resulting in impaired vascular function and increased vascular resistance [112,113] and increase blood pressure over time.

### Alignment with current guidelines

This systematic review and meta-analysis provide regional support to the dietary recommendations set forth by the WHO and the 2014 Nigerian National Nutritional Guidelines for the reduction of hypertension. The recommendations include consuming at least 400 g or five portions of fruits and vegetables per day, limiting salt intalk to less than 5 g per day, reducing consumption of fried and fast foods, restricting red meat consumption to no more than 350–500 g or three portions per week and an avoiding processed meat, keeping total dietary fat intake to less than 30% of the total energy intake and limiting the intake of alcohol to two or less drinks/day for men and one or less drink/day for women in the USA and less than 14 units in the UK [18,114–119]. These findings provide region-specific evidence into dietary factors and hypertension risk and identified six dietary factors (dietary salt, junk food, dietary fat, red meat, and alcohol) as hypertension-inducing dietary factors and fruit and vegetable as protective against hypertension.

### Strengths and limitations

This systematic review and meta-analysis evaluated the association between dietary factors and hypertension across West Africa has notable limitations that could not be avoided: only cross-sectional data were available for this meta-analysis, which cannot confirm a cause-and-effect relationship; all studies relied on participant dietary recall and nonstandardized dietary assessment, which are subject to bias and can potentially affect the accuracy of the dietary data; the majority of the studies in this meta-analysis reported food groups rather than specific foods, which may lead to inaccurate assumptions about specific foods since foods within the same group can impact health differently; and the small number of studies and the limited variation in moderators (e.g. age or BMI) may have limited our ability to uncover some associations. Nonetheless, our study has several notable strengths worth mentioning: this is the first systematic review and meta-analysis to evaluate the association between dietary factors and hypertension across West Africa. This unique focus enables a more compressive overview of the current state of research in West Africa; despite focusing on a specific region of Africa, a large number of studies and participants were included in this meta-analysis, providing a comprehensive understanding of the topic. This inclusion allows for a broad analysis of the impact of diet on hypertension; a diverse selection of dietary factors was identified that reflected foods groups common to healthy and unhealthy patterns enabling a broad analysis of the impact of diet on hypertension; and analyses were critically appraised for risk of bias and power to ensure high-quality.

In conclusion, this systematic review and meta-analysis demonstrated that high consumption of dietary salt, red meat, junk food, dietary fat, and alcohol are associated with an increased likelihood of hypertension in West Africa. In contrast, high fruit and vegetable consumption appears protective. The results of this study will offer regional-specific evidence for developing clinical nutritional assessment tools for clinicians, patients, and researchers and reduce the ever-increasing trend of hypertension in West Africa.

## ACKNOWLEDGEMENTS

The authors sincerely thank the Tertiary Education Trust Fund (TETFund), Nigeria, for funding this study. They also acknowledge the contributions of all the authors and researchers whose work was included in this systematic review and meta-analysis. Finally, they thank all the volunteers in included studies across several countries in West Africa for their dedication and commitment.

This study was funded by the Tertiary Education Trust Fund (TETFund) of Nigeria. MAZ is currently funded by Wellcome Trust (217446/Z/19/Z).

### Conflicts of interest

There are no conflicts of interest.

## Supplementary Material

Supplemental Digital Content

## Supplementary Material

Supplemental Digital Content
