# Supplementary material for: Dietary factors and hypertension risk in West Africa: a systematic review and meta-analysis of observational studies
Source: J Hypertens. 2023 Jul 5;41(9):1376–88. doi: 10.1097/HJH.0000000000003499 (PMC10399948; doi:10.1097/HJH.0000000000003499)
Supplement: Supplemental Digital Content [file jhype-41-1376-s001.pdf]

Dietary factors and hypertension risk in West Africa: A systematic review and meta-analysis of observational studies.

Nimisoere P. Batubo[1], J. Bernadette Moore[1], and Michael A. Zulyniak[1]*
[1]Nutritional Epidemiology Group, School of Food Science and Nutrition, University of Leeds, Leeds, LS2 9JT, United Kingdom.

* Corresponding author: Michael A. Zulyniak, Email: m.a.zulyniak@leeds.ac.uk. Ph. +44(0)1133430685

List of Figures

Figure S1: Funnel plots of 19 studies that reported on the association between fruit and vegetable consumption and hypertension in West Africa.
Figure S2: Forest plot of Subgroup analysis of the moderating effect of (A) mean age, (B) BMI, and study location (C) on the association between fruit and vegetable consumption and hypertension in West Africa.
Figure S3: Bubble plot 19 studies that reported on the association between fruit and vegetable consumption and hypertension.
Figure S4: Funnel plots of 10 studies that reported on the association between junk food consumption and hypertension in West Africa.
Figure S5: Forest plot of Subgroup analysis of the moderating effect of (A) mean age, (B) BMI, and study location (C) on the association between junk food consumption and hypertension in West Africa.
Figure S6 Bubble plot 10 studies that reported on the association between junk food consumption and hypertension.
Figure S7: Funnel plots of 6 studies that reported on the association between red meat consumption and hypertension in West Africa.
Figure S8: Forest plot of Subgroup analysis of the moderating effect of (A) mean age, (B) BMI, and study location (C) on the association between red meat consumption and hypertension in West Africa.
Figure S9: Bubble plot 6 studies that reported on the association between red meat consumption and hypertension.
Figure S10: Funnel plots of 5 studies that reported on the association between dietary fat consumption and hypertension in West Africa.
Figure S11: Forest plot of Subgroup analysis of the moderating effect of (A) mean age, (B) BMI, and study location (C) on the association between dietary fat consumption and hypertension in West Africa.
Figure S12: Bubble plot 5 studies that reported on the association between dietary fat consumption and hypertension. The bubble plot illustrates the relationship of moderating effects of (A) mean age, (B) body mass index (BMI), and (C) study location on the association between red meat consumption and hypertension.
Figure S13: Funnel plots of 5 studies that reported on the association between dietary salt consumption and hypertension in West Africa.
Figure S14: Forest plot of Subgroup analysis of the moderating effect of (A) mean age, (B) BMI, and study location (C) on the association between dietary salt consumption and hypertension in West Africa.
Figure S15: Bubble plot 5 studies that reported on the association between dietary salt consumption and hypertension.
Figure S16: Funnel plots of 5 studies that reported on the association between alcohol consumption and hypertension in West Africa.
Figure S17: Forest plot of Subgroup analysis of the moderating effect of (A) mean age, (B) BMI, and study location (C) on the association between alcohol consumption and hypertension in West Africa.
Figure S18: Bubble plot 5 studies that reported on the association between alcohol consumption and hypertension.

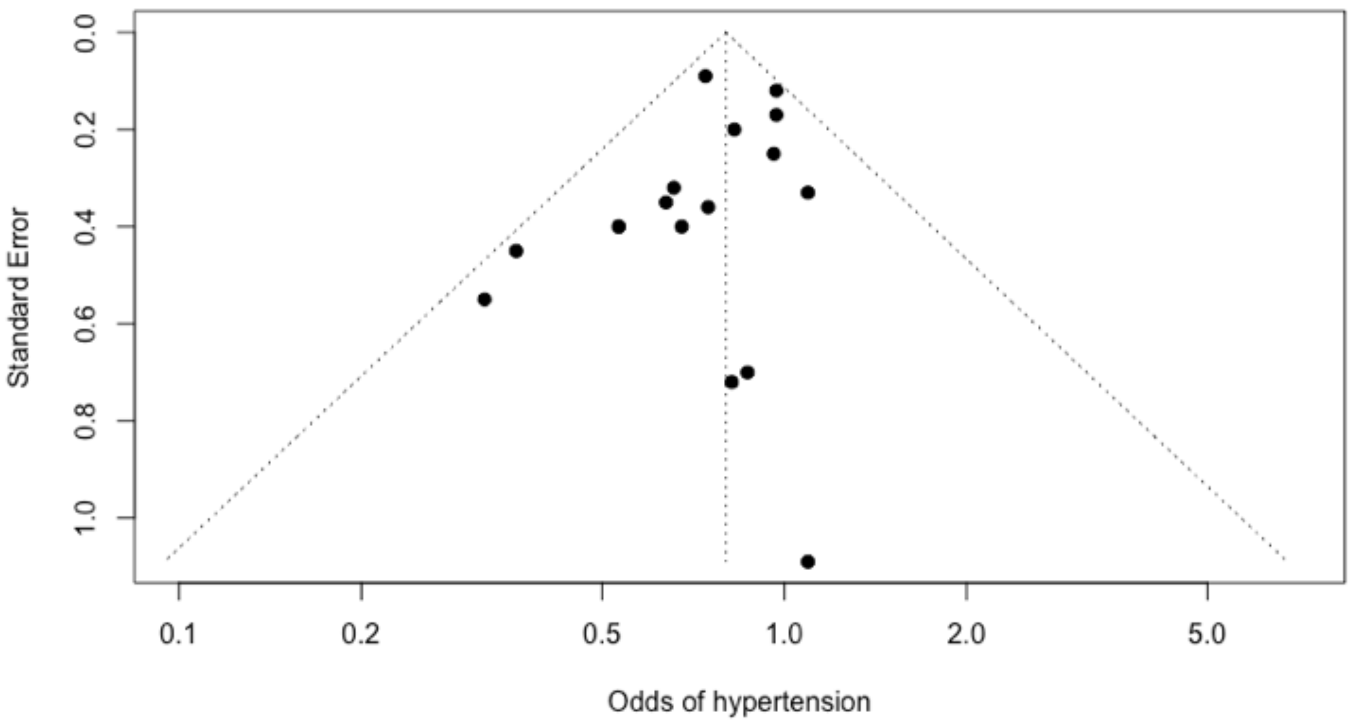

Figure S1: Funnel plot of 17 studies reported the association between fruit and vegetable consumption and hypertension in West Africa with an odds ratio (x-axis) vs standard error (y-axis). No publication bias was demonstrated by the funnel plot, Egger's regression test (p= 0.18), and rank correlation test (p= 0.17) in the meta-analysis.

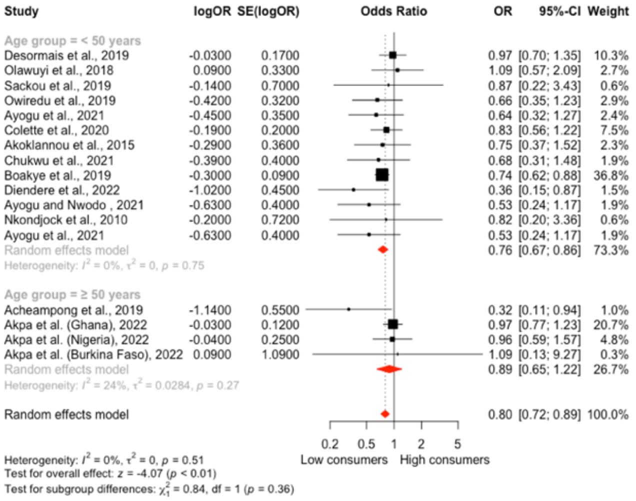

(A)

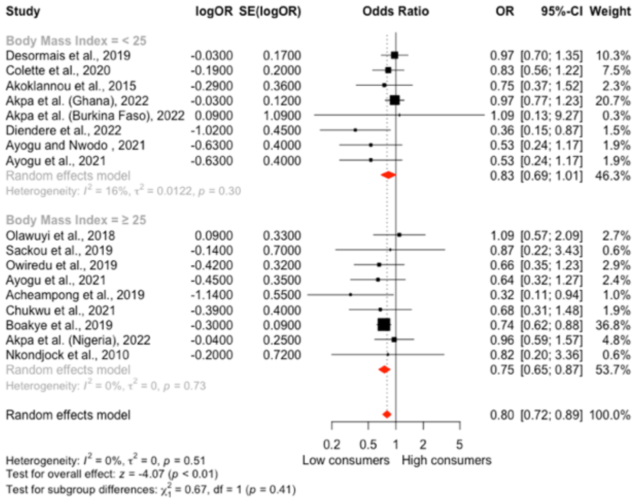

(B)

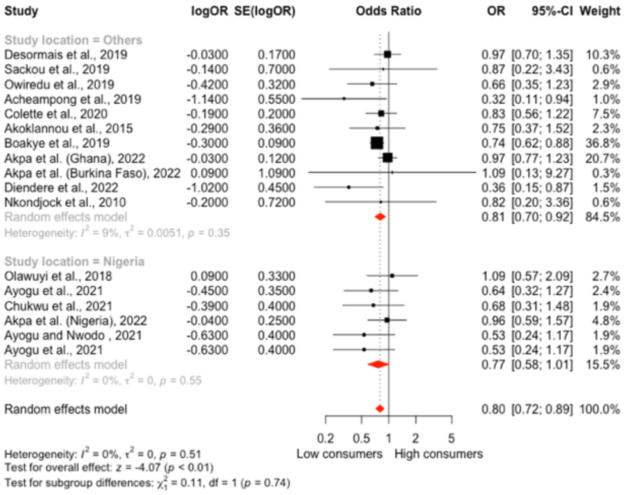

(C)

Figure S2. Forest plot of Subgroup analysis of the moderating effect of (A) mean age, (B) BMI, and study location (C) on the association between fruit and vegetable consumption and hypertension in West Africa. The result in (A) suggests that mean age significantly moderates the effect size and shows a stronger association in individuals < 50 years than those ≥50 years old. No significant moderating effect was observed for BMI and study location in (B) and (C), respectively. CI: Confidence interval, logOR: Treatment effect, OR: Odds ratio, SE (logOR): Standard error.

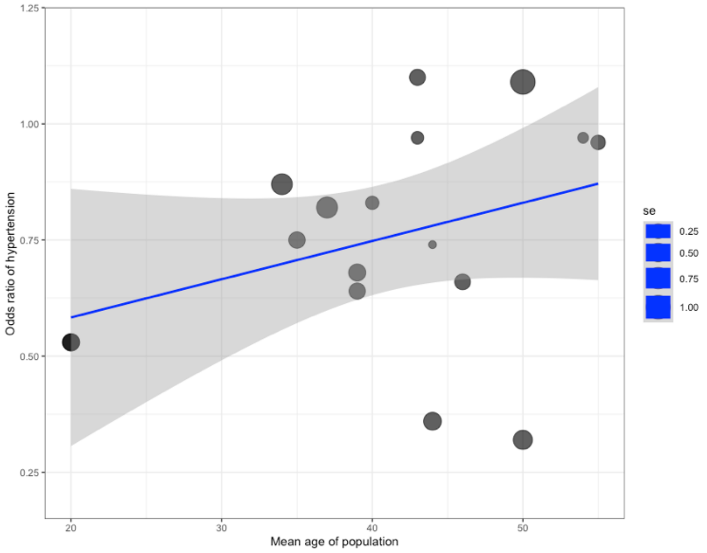

(A)

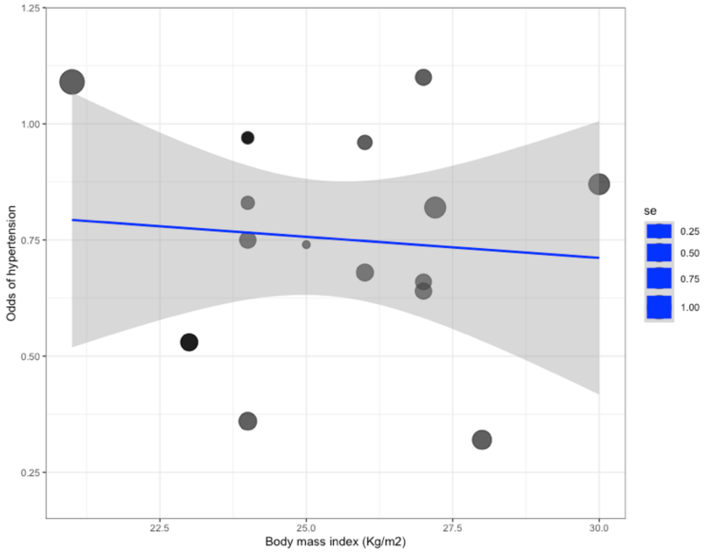

(B)

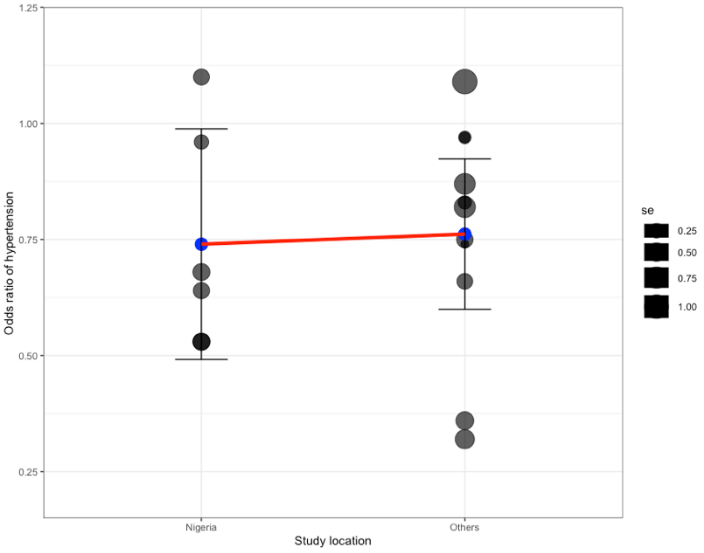

(C)

Figure S3. Bubble plots of 17 studies reported the association between fruit and vegetable consumption and hypertension. The bubble plot illustrates the relationship of moderating effects of (A) mean age, (B) body mass index (BMI), and (C) study location on the association between fruit & vegetable consumption and hypertension. The bubble plot in (A), (B), and (C) suggests that there is no significant moderating effect of the mean age, BMI, and study location on the relationship between fruit consumption and hypertension.

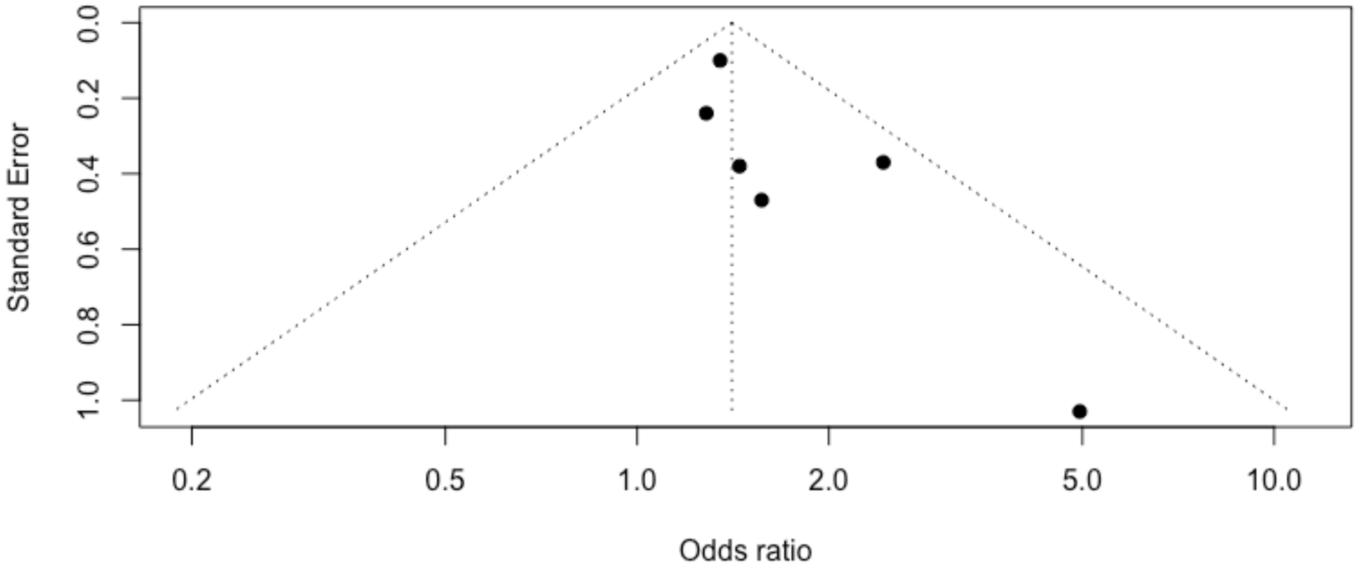

Figure S4. Funnel plot of 5 studies that reported on the association between junk food consumption and hypertension in West Africa with an odds ratio (x-axis) vs standard error (y-axis). No publication bias was demonstrated by the funnel plot, Egger's regression test (p= 0.26), and rank correlation test (p= 0.06) in the meta-analysis.

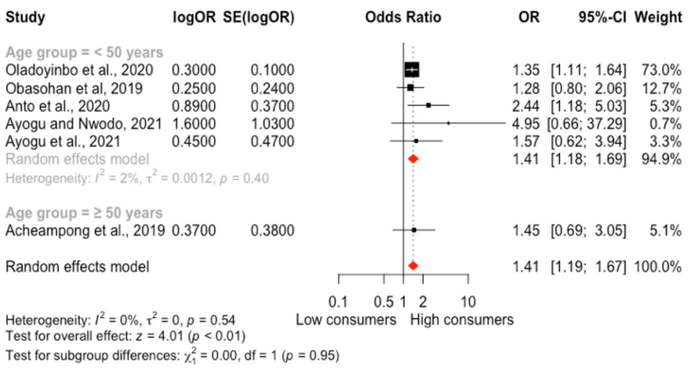

(A)

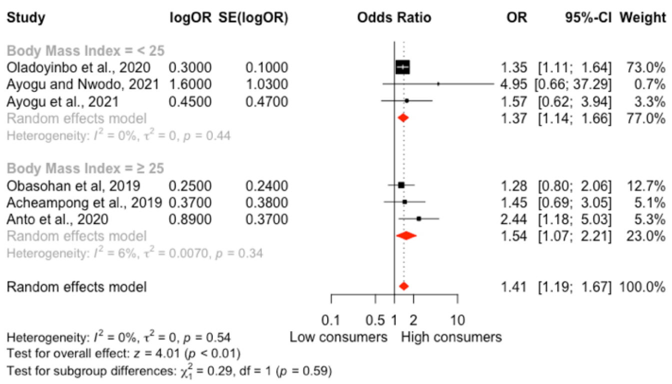

(B)

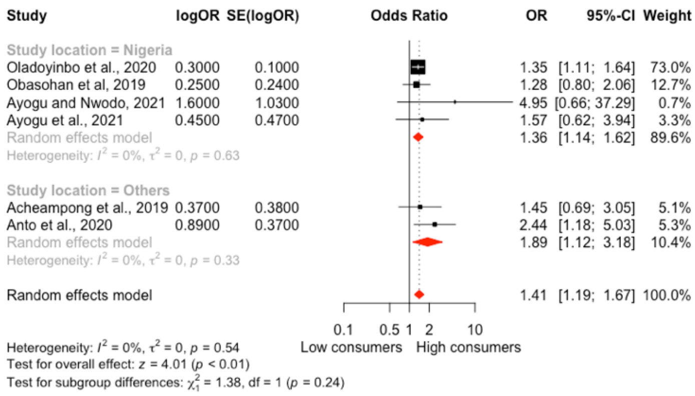

(C)

Figure S5. Forest plots of Subgroup analysis of the moderating effect of (A) mean age, (B) BMI, and study location (C) on the association between junk food consumption and hypertension in West Africa. No significant moderating effect was observed for mean age, BMI, and study location in (A), (B), and (C) on the effect size, respectively. CI: Confidence interval, logOR: Treatment effect, OR: Odds ratio, SE (logOR): Standard error.

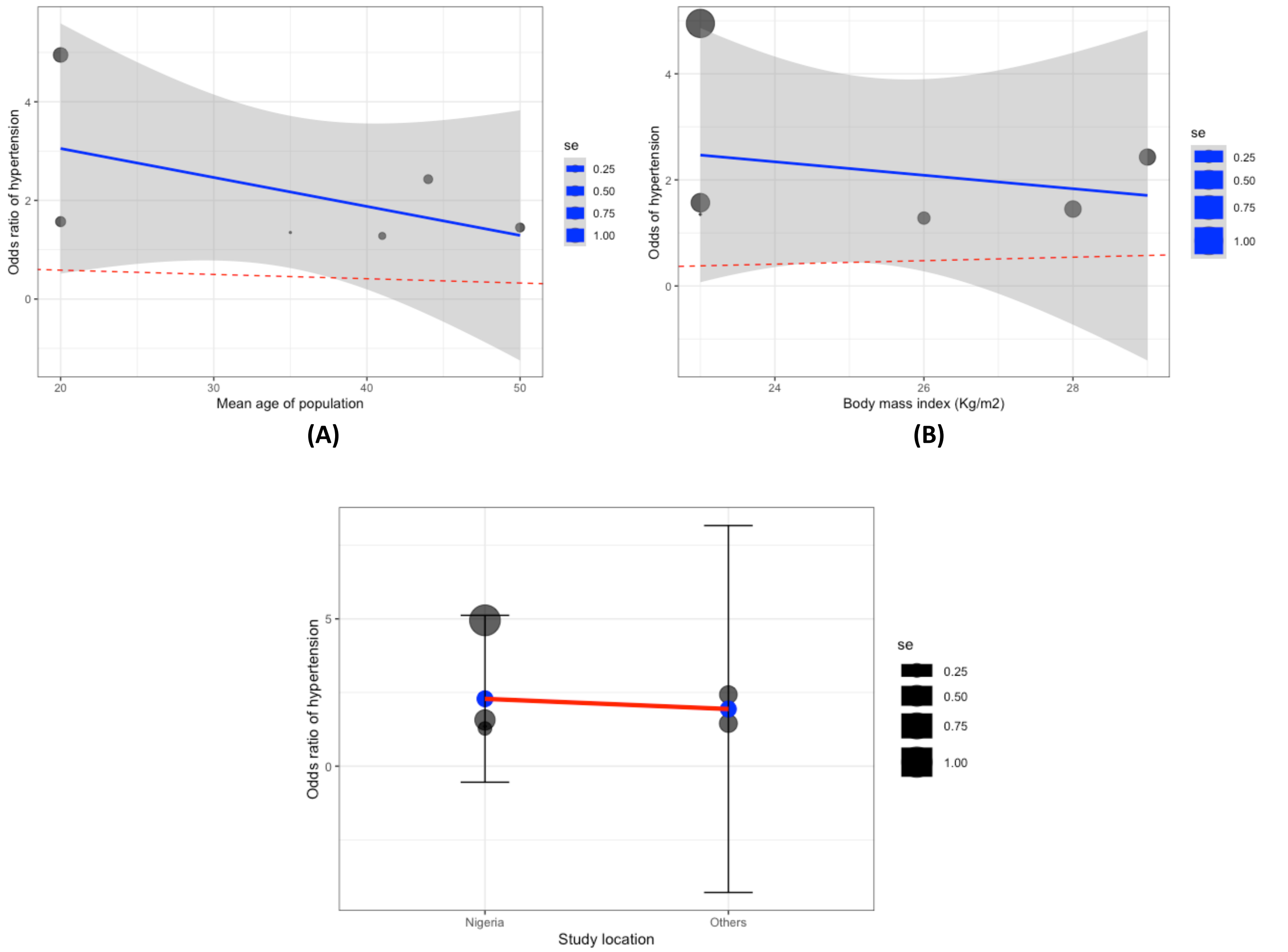

(C)

Figure S6. Bubble plots of 6 studies that reported on the association between junk food consumption and hypertension. The bubble plot illustrates the relationship of moderating effects of (A) mean age, (B) body mass index (BMI), and (C) study location on the association between junk food consumption and hypertension. The bubble plot in (A), (B), and (C) suggests that there is no significant moderating effect of mean age, BMI, and study location on the relationship between fruit consumption and hypertension.

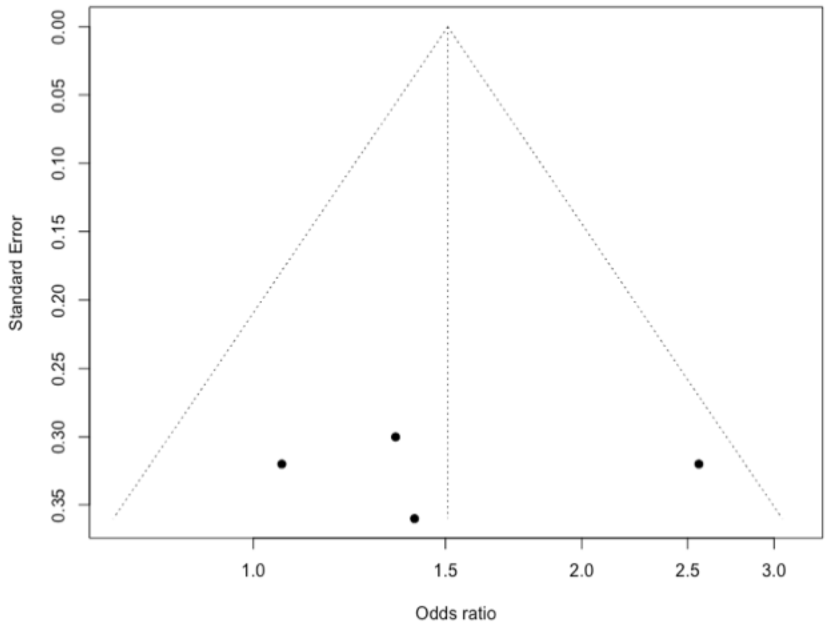

Figure S7. Funnel plot of 4 studies that reported on the association between red meat consumption and hypertension in West Africa with an odds ratio (x-axis) vs standard error (y-axis). No publication bias was indicated by the funnel plot, Egger's regression test (p= 1.00), and rank correlation test (p= 0.72) in the meta-analysis.

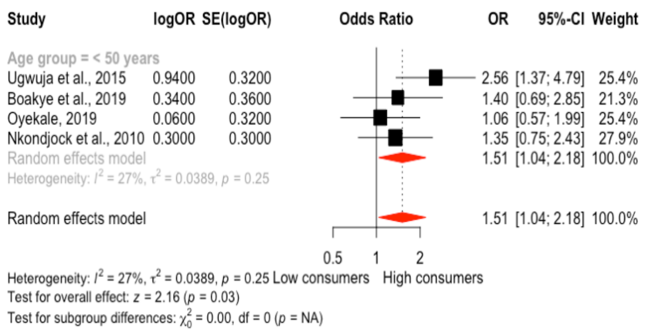

(A)

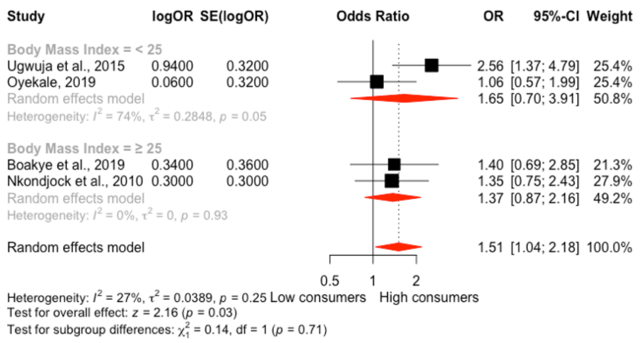

(B)

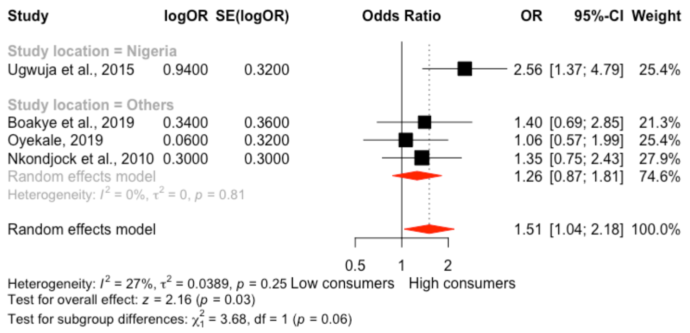

(C)

Figure S8. Forest plot of Subgroup analysis of the moderating effect of (A) mean age, (B) BMI, and study location (C) on the association between red meat consumption and hypertension in West Africa. No significant moderating effect was observed for mean age, BMI, and study location in figures (A), (B), and (C), respectively. CI: Confidence interval, logOR: Treatment effect, OR: Odds ratio, SE (logOR): Standard error.

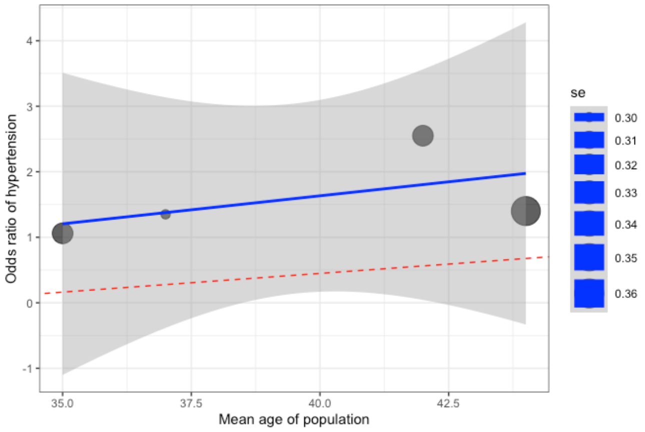

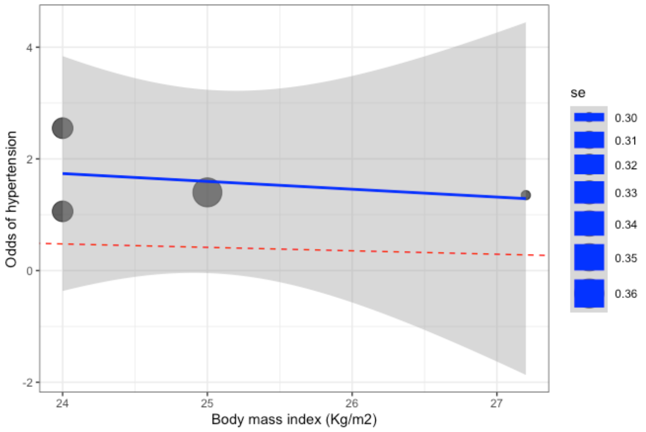

(A) **(B)**

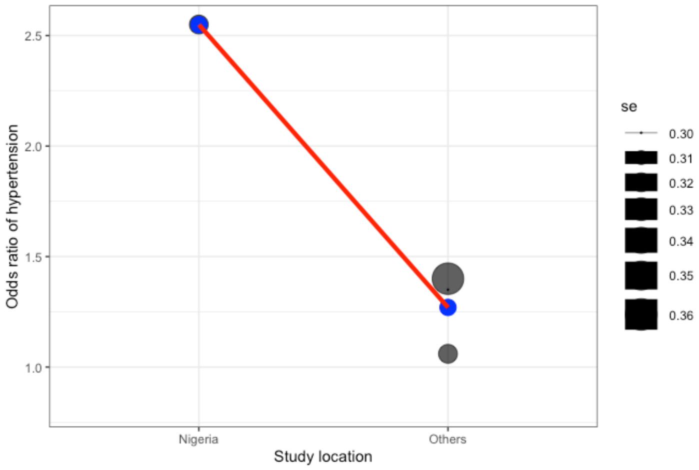

(C)

Figure S9. Bubble plot 4 studies that reported on the association between red meat consumption and hypertension. The bubble plot illustrates the relationship of moderating effects of (A) mean age, (B) body mass index (BMI), and (C) study location on the association between red meat consumption and hypertension. No moderating effect of the mean age, BMI, and study location on the relationship between red meat consumption and hypertension was observed.

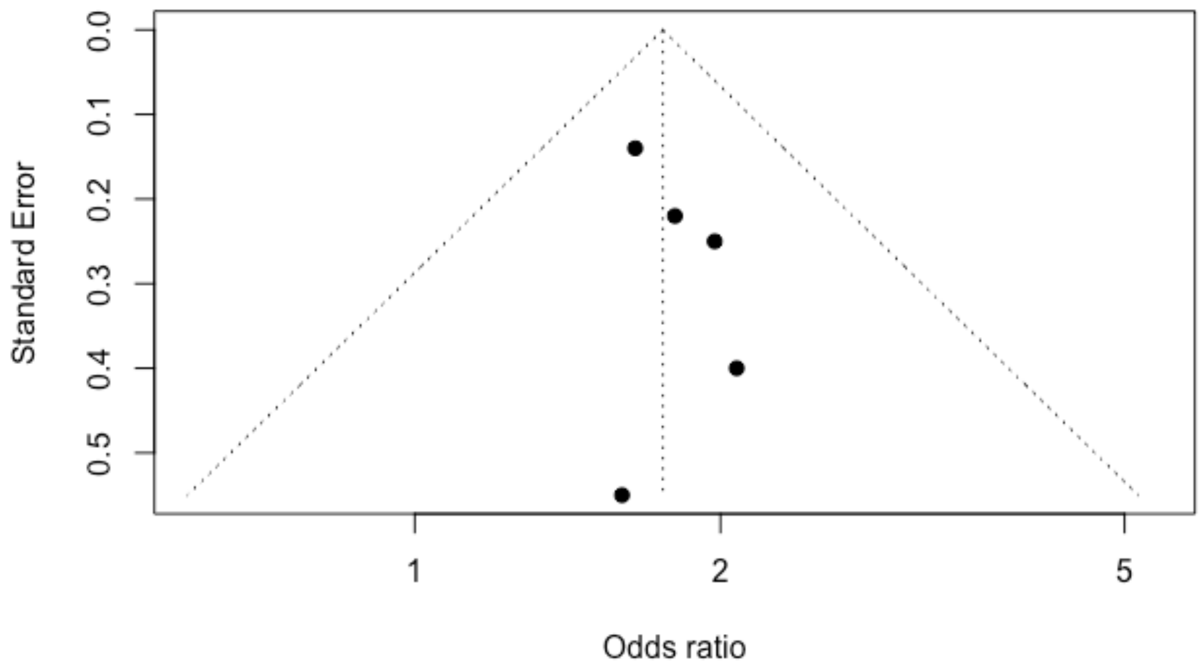

Figure S10. Funnel plots of 5 studies that reported on the association between dietary fat consumption and hypertension in West Africa with an odds ratio (x-axis) vs standard error (y-axis). No publication bias was demonstrated by the funnel plot, Egger's regression test (p= 0.89), and Begg's test (p= 0.48) in the meta-analysis.

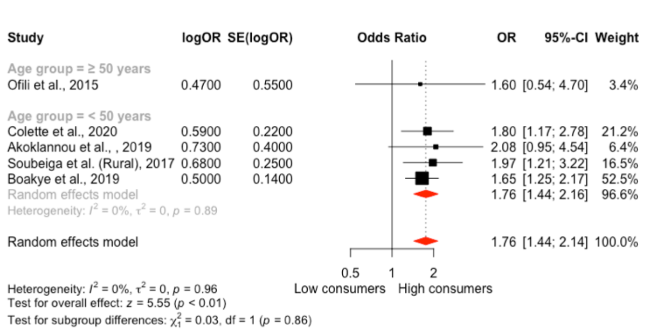

(A)

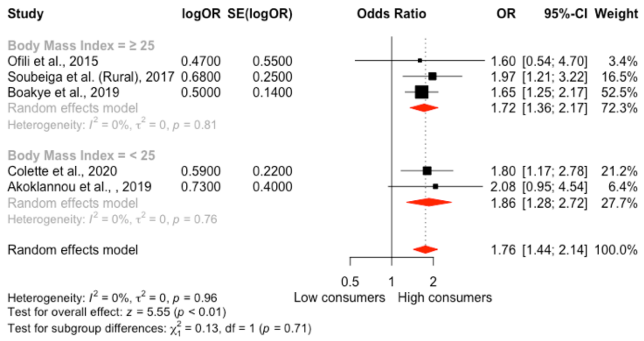

(B)

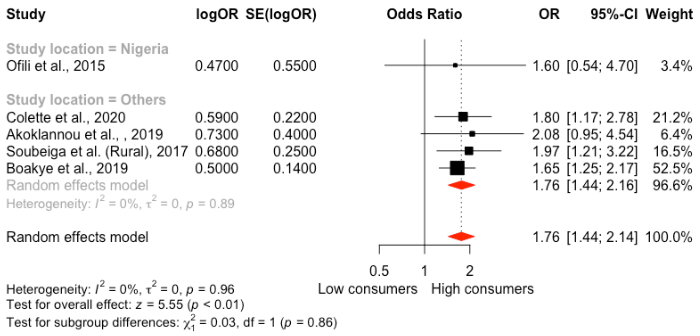

(C)

Figure S11. Forest plot of Subgroup analysis of the moderating effect of (A) mean age, (B) BMI, and study location (C) on the association between dietary fat consumption and hypertension in West Africa. No significant moderating effect was observed for mean age, BMI, and study location in figures (A), (B), and (C) on the effect size, respectively. CI: Confidence interval, logOR: Treatment effect, OR: Odds ratio, SE (logOR): Standard error.

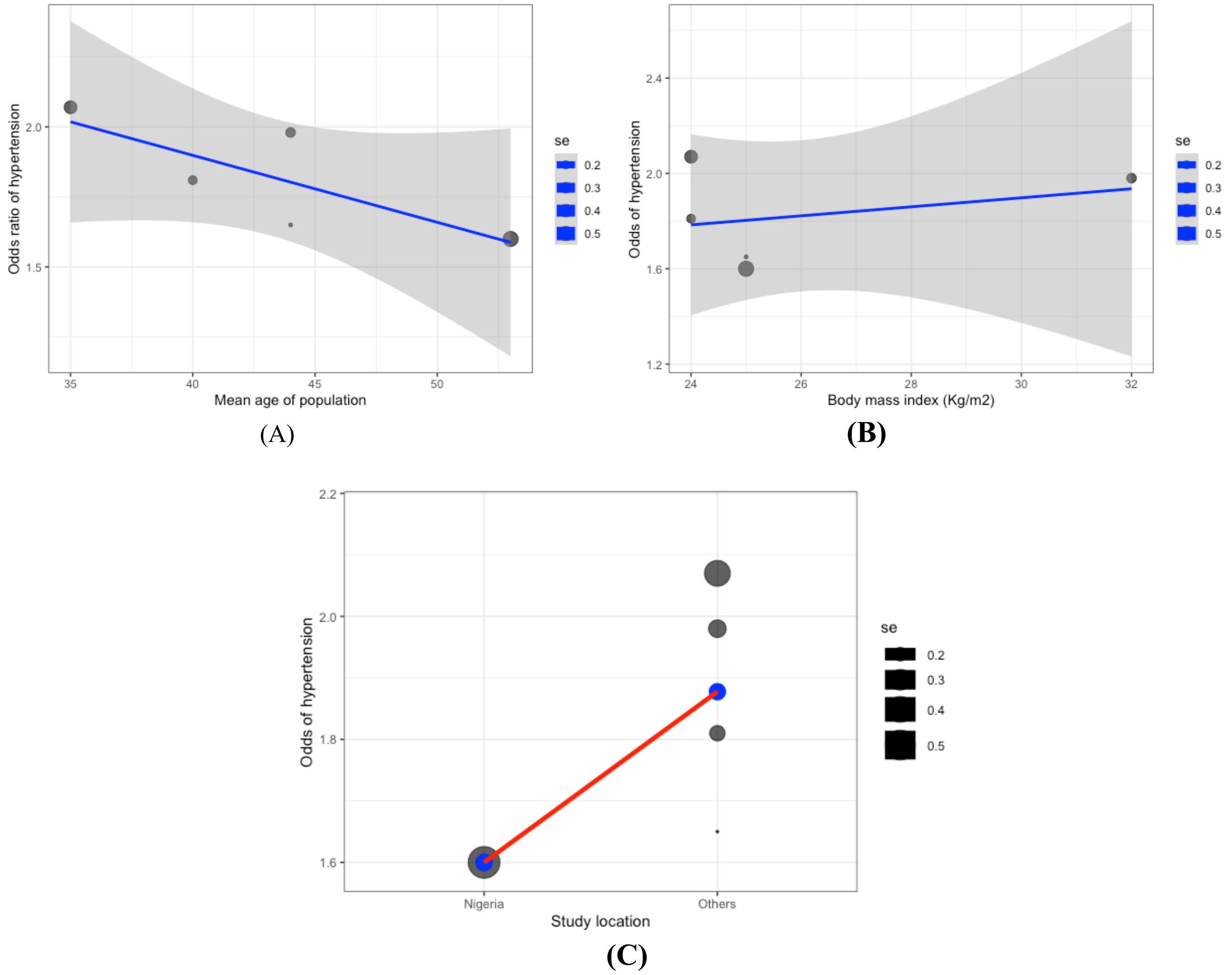

Figure S12. Bubble plot 5 studies that reported on the association between dietary fat consumption and hypertension. The bubble plot illustrates the relationship of moderating effects of (A) mean age, (B) body mass index (BMI), and (C) study location on the association between dietary fat consumption and hypertension. The bubble plot in (A), (B), and (C) suggests that there is no significant moderating effect of the mean age, BMI, and study location on the relationship between dietary fat consumption and hypertension.

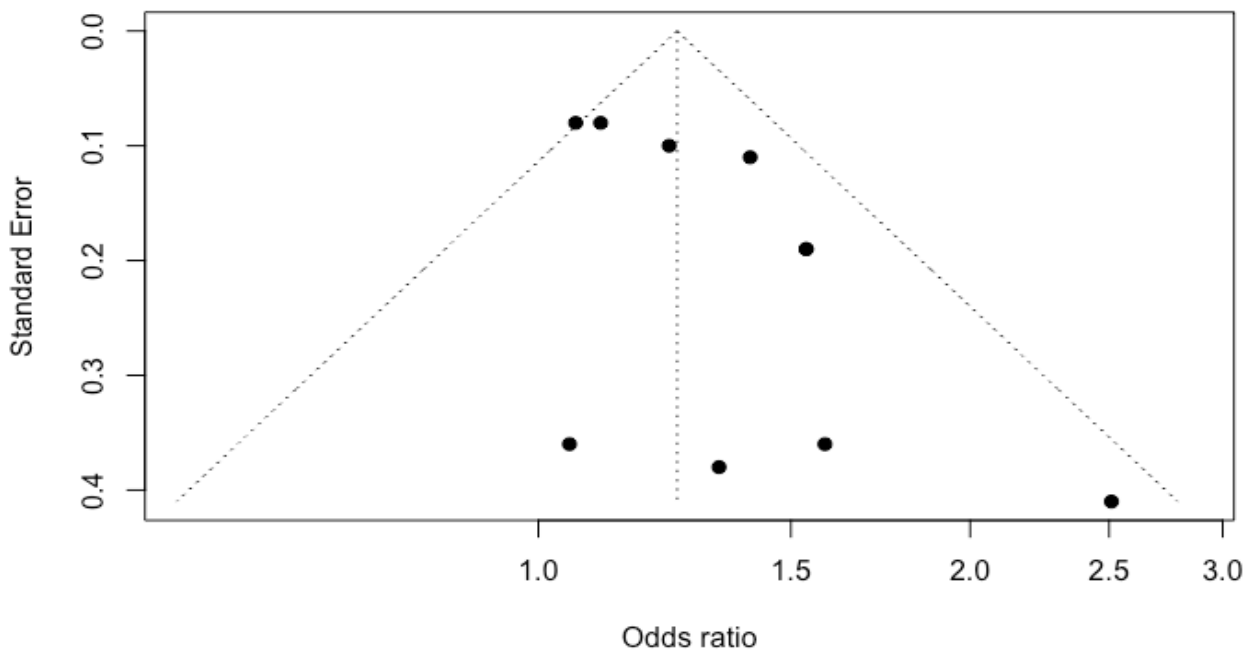

Figure S13. Funnel plot of 10 studies reported the association between dietary salt consumption and hypertension in West Africa with an odds ratio (x-axis) vs standard error (y-axis). No publication bias was demonstrated by the funnel plot, Egger's regression test (p= 0.31), and rank correlation test (p= 0.05) in the meta-analysis.

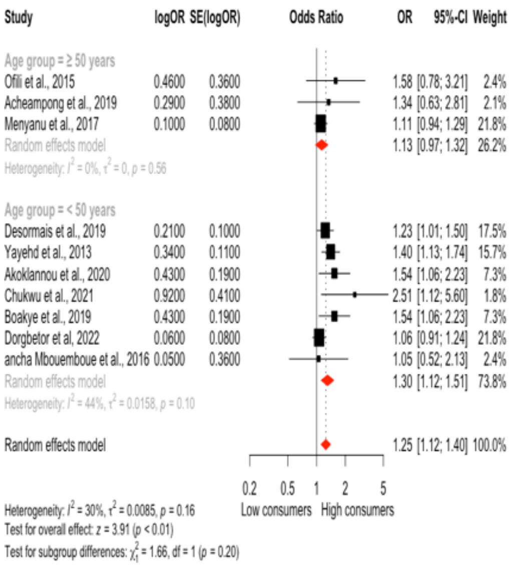

(A)

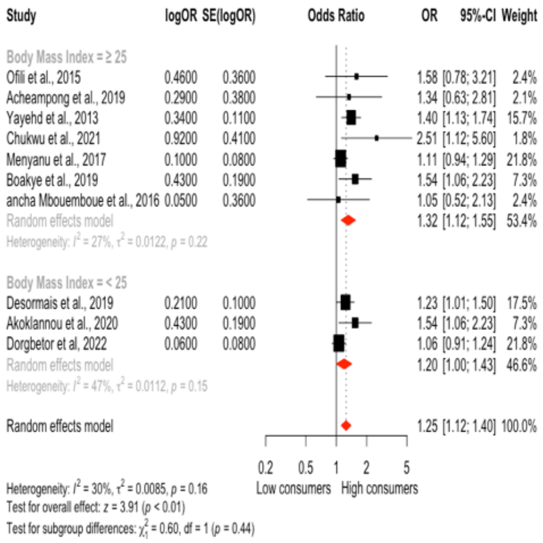

(B)

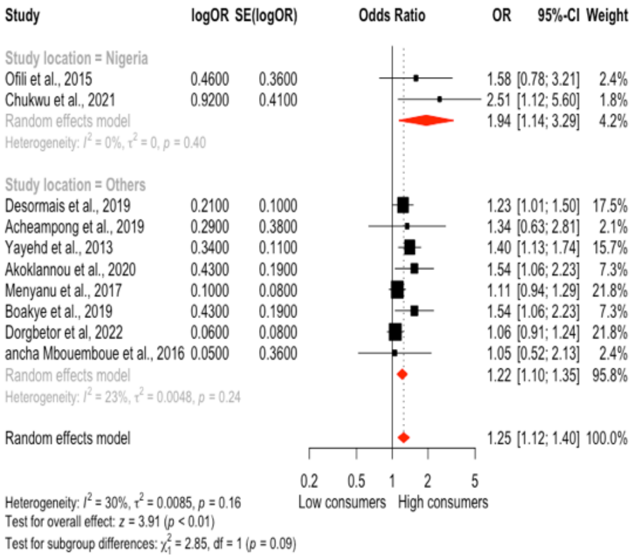

(C)

Figure S14. Forest plot of Subgroup analysis of the moderating effect of (A) mean age, (B) BMI, and study location (C) on the association between dietary salt consumption and hypertension in West Africa. No significant moderating effect was observed for mean age, BMI, and study location in (A), (B), and (C) on the effect size, respectively. CI: Confidence interval, logOR: Treatment effect, OR: Odds ratio, SE (logOR): Standard error.

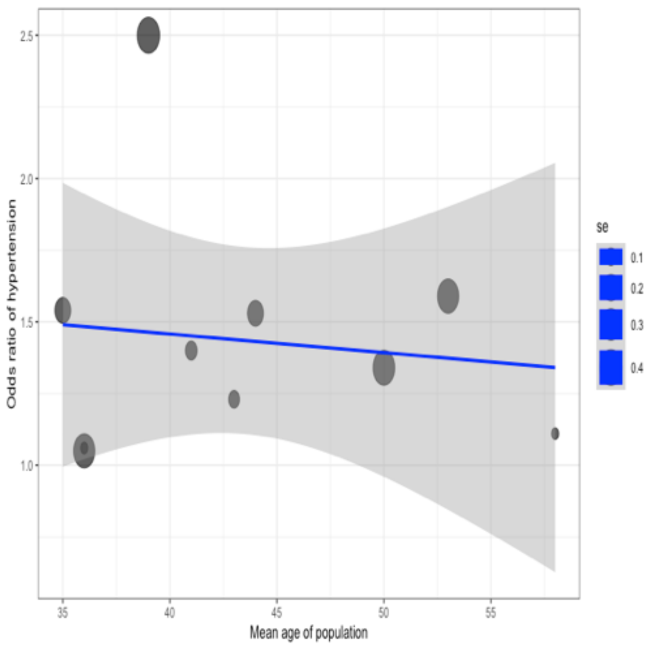

(A)

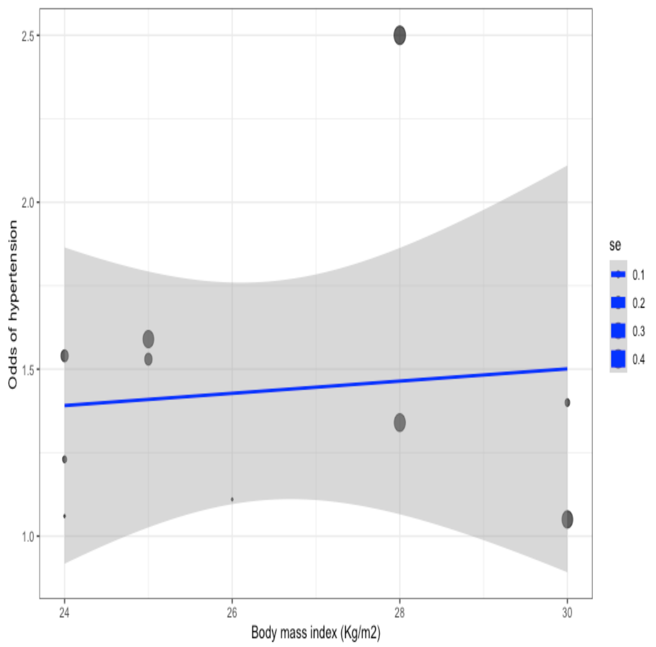

(B)

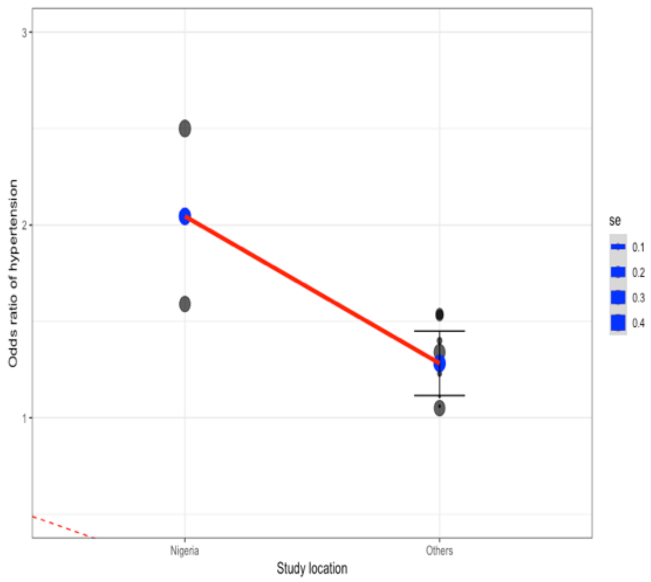

(c)

Figure S15. Bubble plot of 10 studies that reported on the association between dietary salt consumption and hypertension. The bubble plot illustrates the relationship of moderating effects of (A) mean age, (B) body mass index (BMI), and (C) study location on the association between dietary salt consumption and hypertension. The bubble plot in (A), (B), and (C) suggests that there is no significant moderating effect of mean age, BMI, and study location on the relationship between fruit consumption and hypertension.

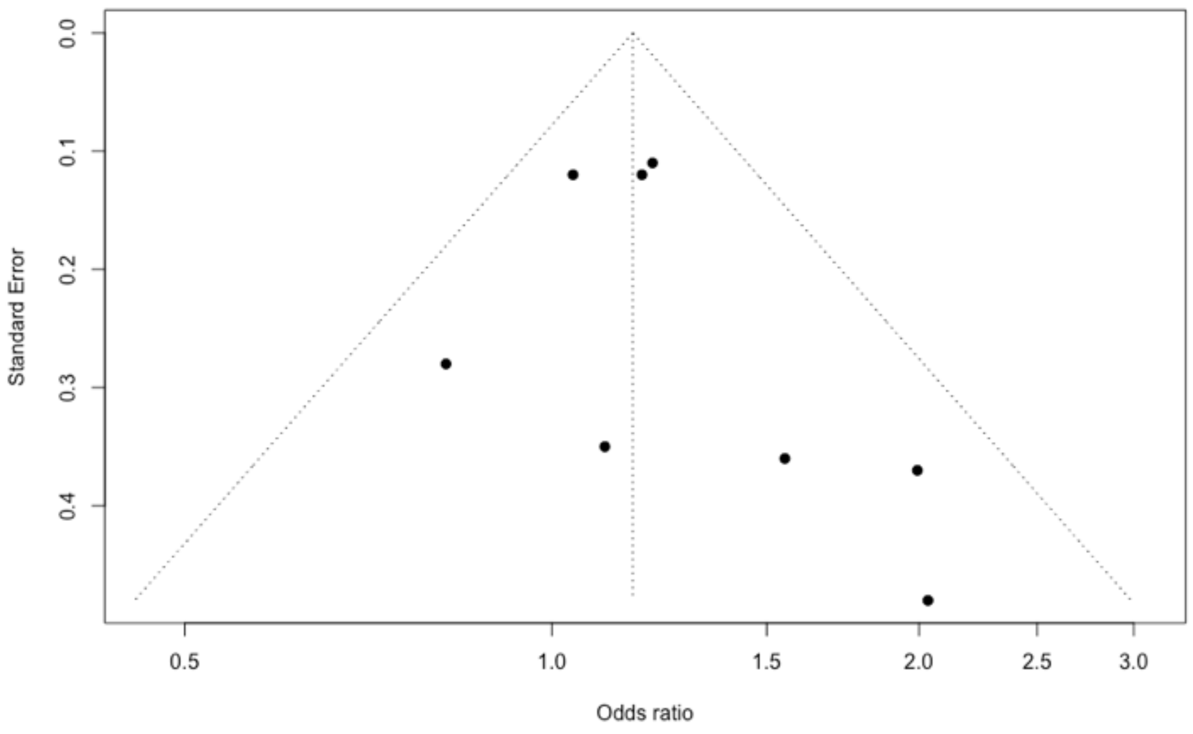

Figure S16. Funnel plots of 8 studies that reported on the association between alcohol consumption and hypertension in West Africa with an odds ratio (x-axis) vs standard error (y-axis). No publication bias was demonstrated by the funnel plot, Egger's regression test (p= 0.46), and Begg's test (p= 0.17) in the meta-analysis.

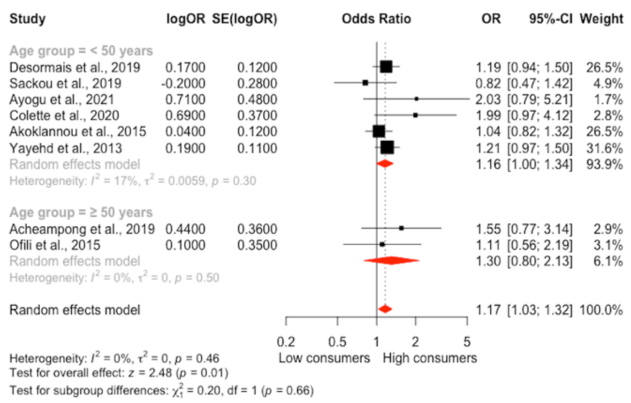

(A)

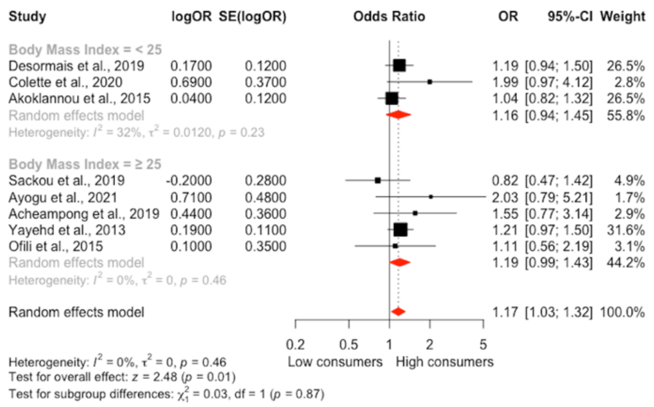

(B)

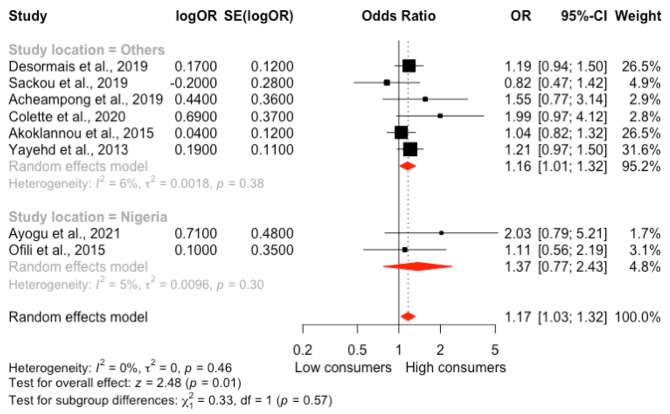

(C)

Figure S17. Forest plot of Subgroup analyses of the moderating effect of (A) mean age, (B) BMI, and study location (C) on the association between alcohol consumption and hypertension in West Africa. No significant moderating effect was observed for mean age, BMI, and study location in (A), (B), and (C) on the effect size, respectively. CI: Confidence interval, logOR: Treatment effect, OR: Odds ratio, SE (logOR): Standard error.

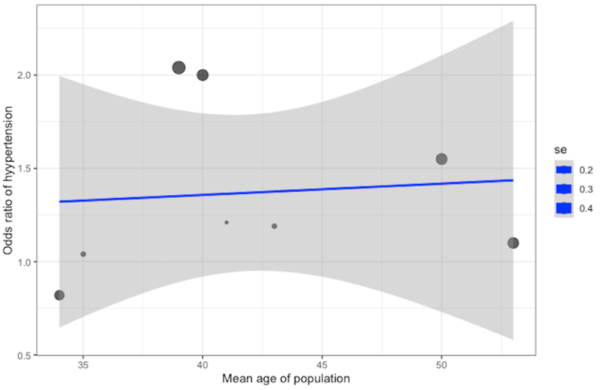

(A)

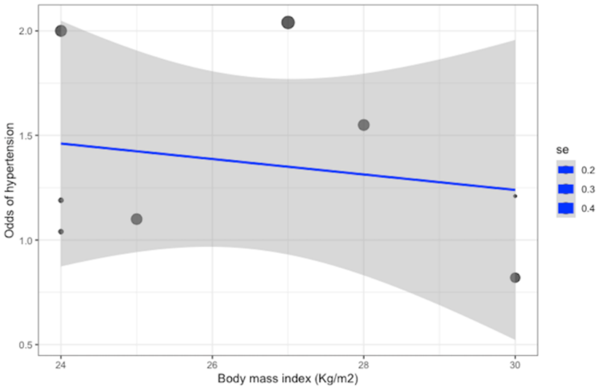

(B)

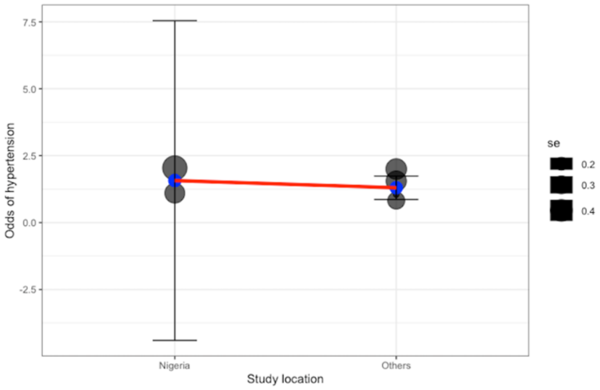

(C)

Figure S18. Bubble plot 5 studies that reported on the association between alcohol consumption and hypertension. The bubble plot illustrates the relationship of moderating effects of (A) mean age, (B) body mass index (BMI), and (C) study location on the association between alcohol consumption and hypertension. The bubble plot in (A), (B), and (C) suggests that there is no significant moderating effect of the mean age, BMI, and study location on the relationship between alcohol consumption and hypertension.
