# Supplementary material for: Dietary factors and hypertension risk in West Africa: a systematic review and meta-analysis of observational studies
Source: J Hypertens. 2023 Jul 5;41(9):1376–88. doi: 10.1097/HJH.0000000000003499 (PMC10399948; doi:10.1097/HJH.0000000000003499)
Supplement: Supplemental Digital Content [file jhype-41-1376-s002.pdf]

Dietary factors and hypertension risk in West Africa: A systematic review and meta-analysis of observational studies.

Nimisoere P. Batubo[1], J. Bernadette Moore[1], and Michael A. Zulyniak[1]*
[1]Nutritional Epidemiology Group, School of Food Science and Nutrition, University of Leeds, Leeds, LS2 9JT, United Kingdom.

* Corresponding author: Michael A. Zulyniak, Email: m.a.zulyniak@leeds.ac.uk. Ph. +44(0)1133430685

List of Tables

Table S1: Search strategy used in PubMed database.
Table S2: Characteristics of the included studies in the meta-analysis investigating the association between dietary factors and odds of hypertension.
Table S3: Quality assessment of studies included in the meta-analysis using the Newcastle-Ottawa Scale adapted for cross-sectional studies for assessing studies in the meta-analysis of the association between dietary factors and hypertension in West Africa.
Table S4: Meta-regression results for moderating effects of mean age, study location, and BMI on the association between fruit and vegetable consumption and hypertension in West Africa.
Table S5: Meta-regression results for moderating effects of mean age, study location, and BMI on the association between junk food consumption and hypertension in West Africa.
Table S6: Meta-regression results for moderating effects of mean age, study location, and BMI on the association between red meat consumption and hypertension in West Africa.
Table S7: Meta-regression results for moderating effects of mean age, study location, and BMI on the association between dietary fat consumption and hypertension in West Africa.
Table S8: Meta-regression results for moderating effects of mean age, study location, and BMI on the association between dietary salt consumption and hypertension in West Africa.
Table S9: Meta-regression results for moderating effects of mean age, study location, and BMI on the association between alcohol consumption and hypertension in West Africa.
Table S10: PRISMA-P (Preferred Reporting Items for Systematic Review and Meta-Analysis Protocols) checklist: for dietary factors and hypertension in West Africa.

Table S1: Search strategy used in PubMed database.

PICO Framework	Concept	Search terms
P- West African nations	1	"Africa, Western"[Mesh] OR “West Africa” OR Benin OR "Burkina Faso" OR "Cape Verde" OR "Cote d'Ivoire" OR “Ivory coast” OR Gambia OR Ghana OR Guinea OR Guinea-Bissau OR Liberia OR Mali OR Mauritania OR Niger OR Nigeria OR Senegal OR Sierra Leone OR Togo OR Cameroon
I – High dietary exposure	2	(("Diet"[Mesh]) OR "Nutrients"[Mesh]) OR "Food"[Mesh] OR Diet* OR nutrition OR nutrient* OR "food habit*" OR "eating habit*" OR lifestyle OR food* OR "dietary pattern*" OR “fatty food*” OR fat* OR DASH OR “Dietary approaches to stop hypertension” OR “table salt*” OR salt* OR egg* OR vegetables OR “fruit and vegetable” OR fruit* OR “healthy diet*” OR “low-fat dairy product*” OR potassium OR sodium OR meat OR starch OR carbohydrate* OR “dietary supplement*” OR sugar* OR alcohol
C- Low dietary exposure		
O- Hypertension/ high blood pressure	3	"Hypertension"[Mesh] OR hypertension OR "high blood pressure" OR "high systolic blood pressure" OR "high diastolic blood pressure" OR SBP OR DBP OR "elevated blood pressure" OR HTN

Table S2. Characteristics of the included studies in the meta-analysis investigating the association between dietary factors and odds of hypertension

S/No	The first author, published year	Study type	Study location	Mean age	Number of Participants	Sex		Exposure		Hypertension		Adjusted covariates	Result
						Male	Female	Dietary Assessment method	Estimated dietary factor	Method of assessment	Number of Cases		(High vs low intake category) OR (95% CI)
1.	Anto, Owiredu [1]	CS	Ghana	44	527	Nil	Nil	Structured questionnaire	Fast food	Measured	204	Age, ethnicity, and family history of hypertension	FF (2.43; 1.18-5.02)
2.	Sackou, Tiade [2]	CS	Côte d’Ivoire	34	360	123	170	WHO Stepwise questionnaire	Fruit & vegetables, alcohol	measured	67	NA	FV (>5) 0.87 (0.22-3.45)<br>Alcohol (0.82; 0.47-1.41)
3.	Ugwuja, Ezenkwa [3]	CS	Nigeria	42	271	86	185	Structured questionnaire	Red meat, fish	measured	62	NA	Red meat: fish [27(11): 240(51)]; 2.55(1.11-5.83)
4.	Oguoma, Nwose [4]	CS	Nigeria	40	417	145	272	WHO Stepwise questionnaire	Fruit & vegetables	measured	34	Age, gender, exposure to second-hand smoke and physical activity status	FV (<5) : (1.06; 1.01–1.13), (>5): 0.94(0.90-0.98)
5.	Olawuyi and Adeoye [5]	CS	Nigeria	43	606	326	280	WHO Stepwise questionnaire	Fruit & Vegetables	measured	201	Age, gender, exposure to second-hand smoke and physical activity status	FV (low): 0.91 (0.48-1.72), (high): 1.10(0.58-2.08)
6.	Acheampong, Nyamari [6]	CS	Ghana	50	216	NA	NA	WHO Stepwise questionnaire	Extra salt<br>Fruit & vegetables<br>Fast food,<br>alcohol	measured	73	age, marital status, education status, employment status, alcohol drinking, extra salt intake, fast foods intake, and body mass index	FV (<5): 3.17 [1.05-9.55], (>5): 0.32(0.11-0.93)<br>Salt: 1.34 [0.64-2.81]<br>FF: (No): 0.69[0.33-1.45]-(High): (1.45(0.69-3.05)<br>Alcohol (1.55; 0.77-3.12)
7.	Obasohan, Okorie [7]	CS	Nigeria	41	476	NA	NA	Food Frequency Questionnaire (FFQ)	Fast Food	measured	208	Age, obesity, fast food, education status, MDA place of work	FF=(>3/week): 1.28(0.80 2.031)
8.	Menyanu, Charlton [8]	CS	Ghana	58	4675	1954	2799	WHO Stepwise questionnaire	Extra salt use	Measured	1444	sex, age, residence, educational level and hypertension prevalence	Salt: 1.11 (0.952–1.299)
9.	Desormais, Amidou [9]	CS	Benin	43	1777	695	1082	WHO Stepwise survey	Fruits and vegetables<br>Salt intake, alcohol	Measured	584	age, sex, country, area, marital status, and previous occupation, tobacco use, BMI, physical activity, diabetes, salt consumption, alcohol consumption, fruits & vegetables	FV (>5): 0.97(0.69-1.36)<br>Salt (1.23 (1.00–1.50)<br>Alcohol (1.19; 0.94-1.53)
10.	Ayogu, Ezeh [10]	CS	Nigeria	42	517	213	287	Interviewer administered questionnaire	Fruit, vegetable, nut, legume, alcohol	Measured	195	Age, sex, educational level, self-percieved health status, obesity, alcohol intake, fruit, vegetable, nuts , legume	F (>5): 0.64 (0.32–1.27)<br>V (>5): 0.82 (0.54–1.24)<br>FV (>5): 0.77(0.54-1.09)<br>Alcohol (2.04; 0.80-5.20)
11.	Ofili, Ncama [11]	CS	Nigeria	53	134	48	86	Questionnaire	Salt, fat, alcohol	Measured	59	Age, marital status, gender, education, family history hypertension, smoking status, alcohol consumption, physical activity, BMI, fruit and vegetable consumption, salt	Salt: 1.59 (0.79, 3.20)<br>Fat: 1.60 (0.54, 4.75)<br>Alcohol (1.10; 0.55-2.20)
12.	Nkondjock and Bizome [12]	CS	Cameroon	37	571	473	98	food frequency questionnaire	Fruit & vegetable<br>Meat (bush meat, red meat)	Measured	223	age, body mass index, rank, vigorous physical activity and total energy intake, the fruit and vegetable	FV (high): 0.82(0.20-2.19)<br>Meat: 1.35 (0.74–2.47)

13.	Pancha Mbouemboue, Derew [13]	CS	Cameroon	36	700	340	360	Semi-structured questionnaire	Salt	Measured	143	Age, marital status, gender, education, family history hypertension, smoking status, alcohol consumption, physical activity, BMI, salt, hyperlipidaemia	Salt (high): 1.05 [0.52–2.07]
14.	Charity E. C, Osaretin A.T. E [14]	CS	Nigeria	39	410	230	180	WHO STEPS questionnaire	Fruit, vegetable, salt intake	Measured	205	Physical activity, fruit and vegetable, current smokers, current alcohol drinkers, salt intake, obesity, diabetes, hypercholesterolemia	FV (>5): 0.15 (0.09, 0.27)<br>Salt<br>Never: 0.400 (0.198–0.81)<br>Often: 2.5 (1.23-5.05)
15.	Oladoyinbo, Abiodun [15]	CS	Nigeria	35	300	192	108	structured questionnaire	Snack, puff puff, fried foods	measured	144	BMI, age, smoking, meal skipping, snacking, physical activity, alcohol,	FAST: 1.35 (0.29, 1.65)
16.	Ayogu, Ezeh [10]	CS	Nigeria	56	517	213	287	Interviewer administered questionnaire	Fruit, vegetable, nut, legume, alcohol	Measured	195	Age, sex, educational level, self-percieved health status, obesity, alcohol intake, fruit, vegetable, nuts , legume	F (>5): 0.64 (0.32–1.27)<br>V (>5): 0.82 (0.54–1.24)<br>FV (>5): 0.77(0.54-1.09)<br>Alcohol (2.04; 0.80-5.20)
17.	Ayogu and Nwodo [16]	CS	Nigeria	20	401	188	213	Interviewer method of questionnaire	Snacks, baked food products, fruits, vegetables	Measured	76	Age, sex, marital status, Socioeconomic variables of interest were occupation and monthly income, smoking, alcohol consumption, sleep duration and exercise, Meal skipping, consumption of snacks, fruits, vegetables and sugar- containing drinks	Snacks= 1.30 (0.08,21.98)<br>FF= 4.95 (0.66, 37.13)<br>FE= 3.15 (0.61, 16.26)<br>Sugar drinks= 1.57(0.62, 3.93)<br>Fruit=0.53(0.24, 1.17)
18.	Akoklannou A. H, Yanogo [17]	CS	Benin	35	717	327	390	WHO Stepwise survey	Salt<br>Fatty food,<br>Fruits and vegetables, alcohol	Measured	238	Sex, age, ethinicity, marital status, salt intake, fatty food consumption, physical activity, fruit and vegetable, alcohol consumption, tobacco consumption, BMI, Glucose level	High salt (1.54; 1.07 - 2.21)<br>Fruit (<5): 1.34(0.66-2.70), (>5): 0.75(0.37-1.52)<br>fat (2.07; 0.94 - 4.54)<br>Alcohol (1.04; 0.83-1.30)
19.	Soubeiga, Millogo [18]	CS	Burkina Faso (Rural)	44	3600	1773	1827	WHO Stepwise approach to Surveillance survey	Butter, lard, margarine, vegetable oil	Measured	553	Age, education, marital status, sex, family history of HBP, smoking, physical activity, fats, BMI, HDL cholesterol	High fats: 1.42 (0.89–2.27)<br>1.98(1.22–3.22)
20.	Yayehd, Damorou [19]		Togo	49	2002	907	1095	Interviewer administered questionnaire	Salt, alcohol	measured	734	Alcohol, Tobacco, salt, obesity, cola, oestrogens	High Salt: 1.4; 1.13–1.72)<br>Alcohol (1.21; 0.97-1.51)
21.	Colette A., Charles S. J. [20]		Benin	40	540	NA	NA	questionnaire	Salt, fat<br>Fruit & vegetable, alcohol	Measured	154	Place of residence, salt, fruit and vegetables, tobacco, physical use, physical activity, obesity, alcohol intake	F (>5): 0.83 [0.56; 1.23]<br>Salt (3.40; 1.76-6.57)<br>Fat (1.81; 1.17-2.81)<br>Alcohol (2.00; 0.97-4.12)
22.	Shokunbi and Ukangwa [21]	CS	Nigeria	20	488	124	219	24-hour dietary recall	Puff-puff, eggs, fruits, vegetables, Beef	Measured	145	NA	Red meat [NO- 343(105); YES- 81(64)] OR=2.87(1.92-4.28)<br>Fried: N0-343(202) vs Yes-94(57): OR-1.08(0.68-1.71)<br>FV: No-343(275), Yes-94(80) (Low): 1.41(0.76-2.65), (High)- 0.71(0.38-1.32)
23.	Kingsley Boakye, Comfort Arthur [22]	CS	Ghana	44	242	104	138	Structured questionnaires	Salt, fruit & vegetable, palm oil, protein (animal, fish and plant)	Measured	90	NA	Salt (High): 1.53(1.05, 2.22)<br>Fruit: (high)= 0.66(0.52, 0.85)<br>Veg: (high)= 0.83 (0.64, 1.08)<br>Red meat=1.40 (0.69, 2.83)<br>Fat: Palm oil=1.65(1.25, 2.19)
24.	Owiredu, Dontoh [23]	CS	Ghana	46	204	80	124	Elaborate pilot-tested questionnaire	Fruit and vegetable	Measured	100	Age, sex, highest educational level, marital status, employment status, physical activity, fruits and vegetable intake, alcohol consumption, and family history of hypertension.	FV (low)= 1.51(0.80-2.84)<br>FV(inverse)= 0.66(0.35, 1.25)
25.	Dorgbetor, Dickson [24]	Survey	Ghana	36	5662	NA	5662	Structured questionnaire	Salted fish	Measured	918	Marital status, age, level of education, wealth index, parity, occupation	Salt (Yes)=1.06 [0.90–1.24]
26.	Diendere, Kabore [25]	Survey	Burkina Faso	44	4187	2257	1930	WHO STEPS survey tool	Fruit and vegetable	Measured	774	living environment, sex, age, marital status, education level, BMI, Physical activity, alcohol use and occupation.	FV (low) = 2.8 (1.2–6.7)<br>FV (inverse)=0.36[0.15, 0.83)
27.	Akpa, Okekunle [26]	Survey	Nigeria	55.4,	3215	1530	1685	FFQ	Vegetables	Measured	1727	Sex, age, education, family history of CVD, ever smoked, ever used alcohol, physical inactivity, BMI, Diabetes, Dyslipidaemia	V(Nig)= 0.96(0.59, 1.57)

28.	Akpa, Okekunle [26]	Survey	Ghana	53.7	3214	1541	1673,	FFQ	Vegetables	Measured	1240	Sex, age, education, family history of CVD, ever smoked, ever used alcohol, physical inactivity, BMI, Diabetes, Dyslipidemia	V(Gha)= 0.97(0.77, 1.23)
29.	Akpa, Okekunle [26]	Survey	Burkina Faso	49.8	2097	1056	1041	FFQ	Vegetables	Measured	345	Sex, age, education, family history of CVD, ever smoked, ever used alcohol, physical inactivity, BMI, Diabetes, Dyslipidemia	V(Bur)= 1.09(0.13, 9.19)
30.	Makinde and Babalola [27]	CS	Nigeria		397	108	289	Food frequency questionnaire	Meat	Measured	39	NA	0.87 (0.37, 1.96)
31.	Oyekale [28]	Survey	Ghana		9367	NA	9367	Food frequency questionnaire	Salted meat	Measured	1244	Age, BMI	β= 0.06, z= 2.03, OR= 1.06 (1.00, 1.12)
	**Total**				**48,290**						**12,223**		

Abbreviations: CS= Cross-sectional study, FF= Fried/fast food, FV= Fruit and vegetable, V= vegetable, β= Beta-coefficient, z=Z-score, Nig= Nigeria, Gha= Ghana, Bur= Burkina Faso, NA= Not available

Table S3: Quality assessment of studies included in the meta-analysis using the Newcastle-Ottawa Scale adapted for cross-sectional studies for assessing studies in the meta-analysis of the association between dietary factors and hypertension in West Africa.

Study	Selection of study groups				Comparability	Outcome		Total score	Study Quality (*9)	Risk of Bias
	Representativeness of the sample (*)	Sample size (*)	Non-Response rate (*)	Ascertainment of the screening/surveillance tool (**)	Potential Confounders (**)	Assessment of Outcome (*)	Statistical test (*)			
Anto, Owiredu [1]	*	*	*	**	**	*	*	********* (9)		Low risk
Sackou, Tiade [2]	*	*	*	**	**	*	*	********* (9)		Low risk
Ugwuja, Ezenkwa [3]	*	*	*	**	-	*	*	******* (7)		Low risk
Oguoma, Nwose [4]	*	*	*	**	-	*	*	********* (9)		Low risk
Olawuyi and Adeoye [5]	*	*	*	**	**	*	*	********* (9)		Low risk
Acheampong, Nyamari [6]	*	*	*	**	**	*	*	********* (9)		Low risk
Obasohan, Okorie [7]	*	*	*	**	**	*	*	********* (9)		Low risk
Menyanu, Charlton [8]	*	*	*	**	**	*	*	********* (9)		Low risk
Desormais, Amidou [9]	*	*	*	**	**	*	*	********* (9)		Low risk
Ofili, Ncama [11]	*	*	*	**	**	*	*	********* (9)		Low risk
Nkondjock and Bizome [12]	*	*	*	**	**	*	*	********* (9)		Low risk
Pancha Mbouemboue, Derew [13]	*	*	*	**	**	*	*	********* (9)		Low risk
Charity E. C, Osaretin A.T. E [14]	*	*	*	**	**	*	*	********* (9)		Low risk
Oladoyinbo, Abiodun [15]	*	*	*	**	**	*	*	********* (9)		Low risk
Ayogu, Ezeh [10]	*	*	*	**	**	*	*	********* (9)		Low risk
Akoklannou A. H, Yanogo [17]	*	*	*	**	**	*	*	********* (9)		Low risk
Soubeiga, Millogo [18]	*	*	*	**	**	*	*	********* (9)		Low risk
Soubeiga, Millogo [18]	*	*	*	**	**	*	*	********* (9)		Low risk
Yayehd, Damorou [19]	*	*	*	**	**	*	*	********* (9)		Low risk
Colette A., Charles S. J. [20]	*	*	*	**	**	*	*	********* (9)		Low risk
Shokunbi and Ukangwa [21]	*	*	*	**		*	*	******* (7)		Low risk
Kingsley Boakye, Comfort Arthur [22]	*	*	*	**		*	*	******* (7)		Low risk
Owiredu, Dontoh [23]	*	*	*	**	**	*	*	********* (9)		Low risk
Dorgbetor, Dickson [24]	*	*	*	**	**	*	*	********* (9)		Low risk
Diendere, Kabore [25]	*	*	*	**	**	*	*	********* (9)		Low risk
Akpa, Okekunle [26]	*	*	*	**	**	*	*	********* (9)		Low risk
Akpa, Okekunle [26]	*	*	*	**	**	*	*	********* (9)		Low risk
Akpa, Okekunle [26]	*	*	*	**	**	*	*	********* (9)		Low risk
Makinde and Babalola [27]	*	*	*	**		*	*	******* (7)		Low risk
Oyekale [28]	*	*	*	**	**	*	*	********* (9)		Low risk

Table S4: Meta-regression results for moderating effects of mean age, study location, and BMI on the association between fruit and vegetable consumption and hypertension in West Africa.

**Moderator**	**n**	**Coefficient (95% CI)**	***p* -Value**	***I^2 (%)**	***R^2 index (%)***	***H^2***
			**Bivariate meta-regression**			
Mean age (years)	17	0.015 (0.001, 0.030)	0.04*	0	0	1.00
Body mass index (kg/m^2)	17	-0.037 (-0.134, 0.059)	0.45	0	0	1.00
Location (Nigeria as ref.)	17	0.051 (-0.245, 0.347)	0.74	0	0	1.00
			**Multivariate meta-regression**			
**Test of moderators**	17	QM (df = 3) = 1.0	0.80	0	0	1.00
Mean age (years)	17	0.015 (-0.016, 0.046)	0.33			
Body mass index (kg/m^2)	17	-0.033 (-0.195, 0.129)	0.67			
location (Nigeria as ref.)	17	-0.081 (-0.690, 0.528)	0.79			

Abbreviations: CI: Confidence interval, H^2: Unaccounted variability/ sampling variability, I^2: Residual heterogeneity/unaccounted variability, n- Number of studies, OR-Odds ratio, R^2: Amount of heterogeneity accounted for. *I^2, and **p*-Value related to the test of moderators. * Significant at 0.05

Table S5: Meta-regression results for moderating effects of mean age, study location, and BMI on the association between junk food consumption and hypertension in West Africa.

**Moderator**	**n**	**Coefficient (95% CI)**	***p* Value**	***I^2* (%) ***	***R^2 index (%)***	***H^2***
			**Bivariate meta-regression**			
Mean age (years)	6	0.001 (-0.031, 0.033)	0.96	2	0	1.00
Body mass index (kg/m^2)	6	0.043 (-0.048, 0.134)	0.35	0	0	1.00
Location (Nigeria as ref.)	6	0.329 (-0.220, 0.877)	0.24	0	0	1.00
			**Multivariate meta-regression**			
**Test of moderators**	6	QM (df = 3) = 0.96	0.74	0	0	1.00
Mean age (years)	6	-0.039 (-0.116, 0.039)	0.33			
Body mass index (kg/m^2)	6	0.077 (-0.342, 0.495)	0.72			
location (Nigeria as ref.)	6	0.400 (-1.573, 2.374)	0.69			

Abbreviations: CI: Confidence interval, H^2: Unaccounted variability/ sampling variability, I^2: Residual heterogeneity/unaccounted variability, n- Number of studies, OR-Odds ratio, R^2: Amount of heterogeneity accounted for. *I^2, and **p*-Value related to the test of moderators. * Significant at 0.05

Table S6: Meta-regression results for moderating effects of mean age, study location, and BMI on the association between red meat consumption and hypertension in West Africa.

**Moderator**	**n**	**Coefficient (95% CI)**	***p* Value**	***I^2* (%) ***	***R^2 index (%)***	***H^2***
			**Bivariate meta-regression**			
Mean age (years)	4	0.059 (-0.038, 0.156)	0.23	16	51	1.18
Body mass index (kg/m^2)	4	-0.062 (-0.394, 0.271)	0.77	48	0	1.92
Location (Nigeria as ref.)	4	-0.711 (-1.438, 0.015)	0.05	0	100	1.00
			**Multivariate meta-regression**			
**Test of moderators**		QM (df = 3) = 2.04	0.56	0	0	1.00
Mean age (years)	4	-0.000 (-0.070, 0.070)	0.99			
Body mass index (kg/m^2)	4	0.060 (-0.411, 0.531)	0.80			
location (Nigeria as ref.)	4	-0.843 (-2.168, 0.481)	0.21			

Abbreviations: CI: Confidence interval, H^2: Unaccounted variability/ sampling variability, I^2: Residual heterogeneity/unaccounted variability, n- Number of studies, OR-Odds ratio, R^2: Amount of heterogeneity accounted for. *I^2, and *p-Value related to the test of moderators. * Significant at 0.05

Table S7: Meta-regression results for moderating effects of mean age, study location, and BMI on the association between dietary fat consumption and hypertension in West Africa.

**Moderator**	**n**	**Coefficient (95% CI)**	***p* Value**	***I^2* (%) ***	***R^2 index (%)***	***H^2***
			**Bivariate meta-regression**			
Mean age (years)	5	-0.015 (-0.077, 0.048)	0.64	0	0	1.00
Body mass index (kg/m^2)	5	0.016 (-0.057, 0.088)	0.67	0	0	1.00
Location (Nigeria as ref.)	5	0.096 (-1.001, 1.193)	0.86	0	0	1.00
			**Multivariate meta-regression**			
**Test of moderators**		QM (df = 3) = 0.16	0.98	0	0	1.00
Mean age (years)	5	-0.029 (-0.193, 0.136)	0.74			
Body mass index (kg/m^2)	5	0.027 (-0.142, 0.193)	0.76			
location (Nigeria as ref.)	5	-0.226 (-2.703, 2.252)	0.56			

Abbreviations: CI: Confidence interval, H^2: Unaccounted variability/ sampling variability, I^2: Residual heterogeneity/unaccounted variability, n- Number of studies, OR-Odds ratio, R^2: Amount of heterogeneity accounted for. *I^2, and **p*-Value related to the test of moderators. * Significant at 0.05

Table S8: Meta-regression results for moderating effects of mean age, study location, and BMI on the association between dietary salt consumption and hypertension in West Africa.

**Moderator**	**n**	**Coefficient (95% CI)**	***p* Value**	***I^2 (%)**	***R^2 index (%)***	***H^2***
			**Bivariate meta-regression**			
Mean age (years)	10	-0.004 (-0.019, 0.012)	0.63	36	0	1.58
Body mass index (kg/m^2)	10	0.022 (-0.027, 0.072)	0.38	28	4	1.38
Location (Nigeria as ref.)	10	-0.468 (-1.015, 0.079)	0.09	19	52	1.23
			**Multivariate meta-regression**			
**Test of moderators**	10	QM (df = 3) = 1.25	0.74	0	0	1.00
Mean age (years)	10	-0.006 (-0.036, 0.025)	0.72			
Body mass index (kg/m^2)	10	0.018 (-0.091, 0.127)	0.74			
location (Nigeria as ref.)	10	-0.470 (-1.370, 0.430)	0.31			

Abbreviations: CI: Confidence interval, H^2: Unaccounted variability/ sampling variability, I^2: Residual heterogeneity/unaccounted variability, n- Number of studies, OR-Odds ratio, R^2: Amount of heterogeneity accounted for. *I^2, and **p*-Value related to the test of moderators. * Significant at 0.05

Table S9: Meta-regression results for moderating effects of mean age, study location, and BMI on the association between alcohol consumption and hypertension in West Africa.

**Moderator**	**n**	**Coefficient (95% CI)**	***p* Value**	**I^2 (%) ***	***R^2 index (%)***	***H^2***
			**Bivariate meta-regression**			
Mean age (years)	8	0.017 (-1.653, 0.576)	1.22	0	0	1.00
Body mass index (kg/m^2)	8	0.000 (-0.050, 0.050)	0.99	10	0	1.12
Location (Nigeria as ref.)	8	-0.167 (-0.741, 0.407)	0.57	6	0	1.07
			**Multivariate meta-regression**			
**Test of moderators**		QM (df = 3) = 0.30	0.96	0	0	1.00
Mean age (years)	8	0.012 (-0.053, 0.076)	0.72			
Body mass index (kg/m^2)	8	-0.008 (-0.124, 0.108)	0.89			
location (Nigeria as ref.)	8	-0.102 (-1.152, 0.949)	0.85			

Abbreviations: CI: Confidence interval, H^2: Unaccounted variability/ sampling variability, I^2: Residual heterogeneity/unaccounted variability, n- Number of studies, OR-Odds ratio, R^2: Amount of heterogeneity accounted for. *I^2, and **p*-Value related to the test of moderators. * Significant at 0.05

Table S10: PRISMA-P checklist: for dietary factors and hypertension in West Africa.

Section and topic	Item No	Checklist item	Reported on page #
**TITLE**			
Identification	1	Identify the report as a protocol of a systematic review and meta-analysis	**1**
**AUTHORS**			
Contact	2	Provide name, institutional affiliation, e-mail address of all protocol authors; provide physical mailing address of corresponding author	**1**
**ABSTRACT**			
Structured summary	3	Provide a structured summary including, as applicable: background; objectives; data sources; study eligibility criteria, participants, and interventions; study appraisal and synthesis methods; results; limitations; conclusions and implications of key findings; systematic review registration number	**2**
**INTRODUCTION**			
Rationale	5	Describe the rationale for the review in the context of what is already known	**3**
Objectives	6	Provide an explicit statement of the question(s) the review will address with reference to participants, interventions, comparators, and outcomes (PICO)	**3**
**METHODS**			
Protocol and registration	7	Indicate if a review protocol exists, if and where it can be accessed (e.g., Web address), and, if available, provide registration information including registration number.	**5**
Eligibility criteria	8	Specify the study characteristics (such as PICO, study design, setting, time frame) and report characteristics (such as years considered, language, publication status) to be used as criteria for eligibility for the review	**5**
Information sources	9	Describe all intended information sources (such as electronic databases, contact with study authors, trial registers or other grey literature sources) with planned dates of coverage	**5**
Search strategy	10	Present draft of search strategy to be used for at least one electronic database, including planned limits, such that it could be repeated	**5, Table S1**
Data management	11	Describe the mechanism(s) that will be used to manage records and data throughout the review	**6**
Study selection process	12	State the process that will be used for selecting studies (such as two independent reviewers) through each phase of the review (that is, screening, eligibility, and inclusion in meta-analysis)	**6**
Data collection process	13	Describe planned method of extracting data from reports (such as piloting forms, done independently, in duplicate), any processes for obtaining and confirming data from investigators	**6**
Data items	14	List and define all variables for which data will be sought (such as PICO items, funding sources), any pre-planned data assumptions and simplifications	**5**
Risk of bias in individual studies	15	Describe anticipated methods for assessing risk of bias of individual studies, including whether this will be done at the outcome or study level, or both; state how this information will be used in data synthesis	**7**
Summary measures	16	State the principal summary measures (e.g., risk ratio, difference in means).	**6**
Synthesis of results	17	Describe the methods of handling data and combining results of studies, if done, including measures of consistency (e.g., I2) for each meta-analysis.	**7**
Risk of bias across studies	18	Specify any assessment of risk of bias that may affect the cumulative evidence (e.g., publication bias, selective reporting within studies).	**7**
Additional analyses	19	Describe methods of additional analyses (e.g., sensitivity or subgroup analyses, meta-regression), if done, indicating which were pre-specified.	**8**
Data synthesis	20	Describe criteria under which study data will be quantitatively synthesised.<br>If data are appropriate for quantitative synthesis, describe planned summary measures, methods of handling data and methods of combining data from studies, including any planned exploration of consistency (such as I^2, Kendall's τ)<br>Describe any proposed additional analyses (such as sensitivity or subgroup analyses, meta-regression)<br>If quantitative synthesis is not appropriate, describe the type of summary planned	**8-9**
**RESULTS**			
Literature search/study selection	21	Give numbers of studies screened, assessed for eligibility, and included in the review, with reasons for exclusions at each stage, ideally with a flow diagram.	**9, Figure 1**
Study characteristics	22	For each study, present characteristics for which data were extracted (e.g., study size, PICOS, follow-up period) and provide the citations.	**9, Table 1, S2**
Risk of bias within studies	23	Present data on risk of bias of each study and, if available, any outcome level assessment (see item 12).	**Table S3**
Results of individual studies	24	For all outcomes considered (benefits or harms), present, for each study: (a) simple summary data for each intervention group (b) effect estimates and confidence intervals, ideally with a forest plot.	**Table 1, S2**
Synthesis of results	25	Present results of each meta-analysis done, including confidence intervals and measures of consistency.	**10-13**
Studies quality	26	Present results of any assessment of risk of bias across studies (see Item 15).	**13, Table S3**
Additional analysis	27	Give results of additional analyses, if done (e.g., sensitivity or subgroup analyses, meta-regression [see Item 16]).	**Figure S3- 18**

**DISCUSSION**			
Summary of evidence	28	Summarize the main findings including the strength of evidence for each main outcome; consider their relevance to key groups (e.g., healthcare providers, users, and policy makers).	**14-19**
Limitations	29	Discuss limitations at study and outcome level (e.g., risk of bias), and at review-level (e.g., incomplete retrieval of identified research, reporting bias).	**19-20**
Conclusions	30	Provide a general interpretation of the results in the context of other evidence, and implications for future research.	**20**
**SUPPORT**			
Sponsor	4	Provide name for the review funder and/or sponsor	**1**
